# Supplementary material for: DNA Methylation Dynamics of Human Hematopoietic Stem Cell Differentiation
Source: Cell Stem Cell. 2016 Dec 1;19(6):808–22. doi: 10.1016/j.stem.2016.10.019 (PMC5145815; doi:10.1016/j.stem.2016.10.019)
Supplement: Document S1. Supplemental Experimental Procedures and Figures S1–S7 [file mmc1.pdf]

Cell Stem Cell, Volume *19*

Supplemental Information

DNA Methylation Dynamics of Human Hematopoietic Stem Cell Differentiation

Matthias Farlik, Florian Halbritter, Fabian Müller, Fizzah A. Choudry, Peter Ebert, Johanna Klughammer, Samantha Farrow, Antonella Santoro, Valerio Ciaurro, Anthony Mathur, Rakesh Uppal, Hendrik G. Stunnenberg, Willem H. Ouwehand, Elisa Laurenti, Thomas Lengauer, Mattia Frontini, and Christoph Bock

Supplemental Figures

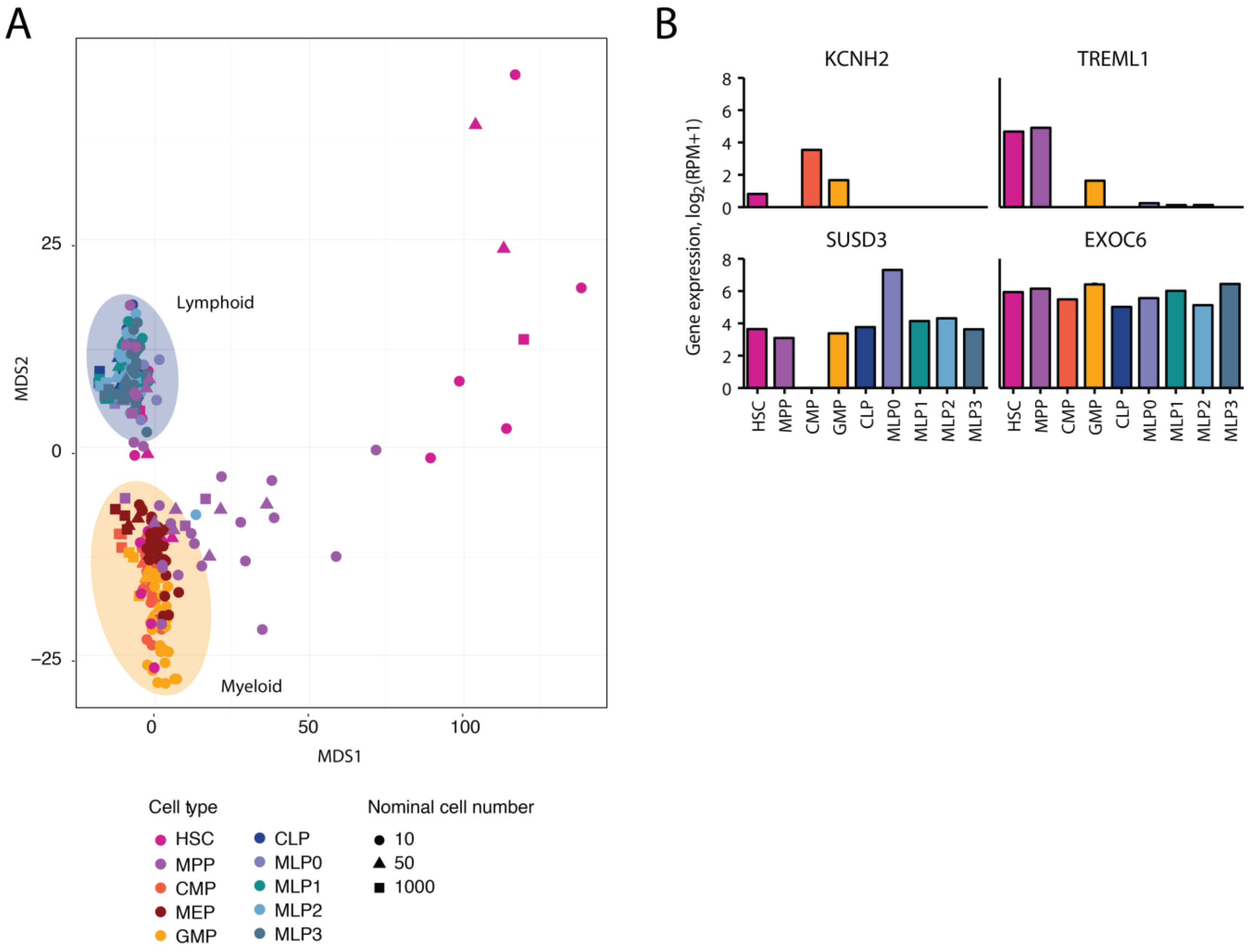

Figure S1. DNA methylation and gene expression profiles of blood stem and progenitor cells

A) Unsupervised multidimensional scaling (MDS) analysis of DNA methylation profiles for 10-cell, 50-cell, and 1,000-cell samples of hematopoietic stem and progenitor cell types sorted from peripheral blood. DNA methylation levels were aggregated at region level based on the BLUEPRINT Regulatory Build. The analysis results are dominated by two compact clusters comprising lymphoid and myeloid cells, while HSC and MPP profiles are separated and more dispersed. The number of cells in each pool did not have a strong effect on the grouping.

B) Gene expression levels of KCNH2, TREML1, SUSD3, and EXOC6 in the indicated stem and progenitor cell types measured by RNA-seq.

Related to Figure 1.

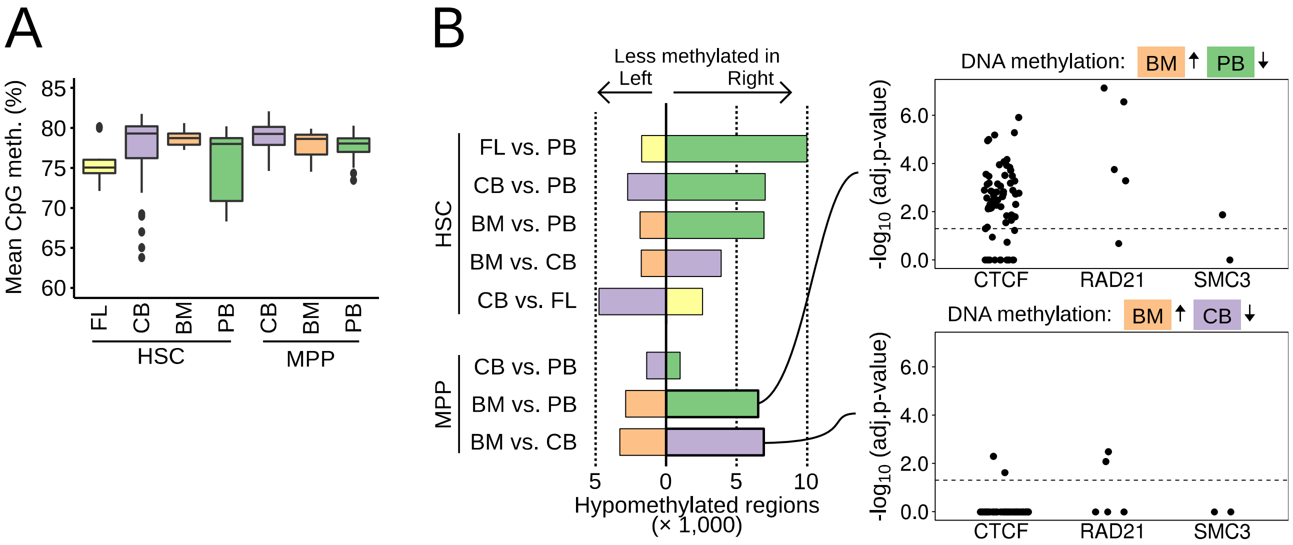

Figure S2. DNA methylation differences for HSCs and MPPs isolated from different sources

A) Distribution of average CpG methylation levels for HSCs (left) and MPPs (right) isolated from different sources. FL: fetal liver, CB: cord blood, BM: bone marrow, PB: peripheral blood.

B) Enrichment of CTCF, RAD21, and SMC3 binding sites for regions with lower DNA methylation in peripheral blood-derived MPPs than in bone-marrow-derived MPPs (left), or with lower DNA methylation in cord blood-derived MPPs than in bone-marrow-derived MPPs (right). Enrichment was determined using LOLA (Sheffield and Bock, 2016). Each dot represents one ChIP-seq dataset, and the dashed line corresponds to a significance threshold of 0.05 on the adjusted p-value calculated by LOLA using Fisher's exact test. Enrichment p-values were high for comparisons that involved peripheral blood-derived HSCs (**Figure 2C**) and peripheral blood-derived MPPs (top right), while they were lower in other comparisons of similar size (bottom right).

Related to Figure 2.

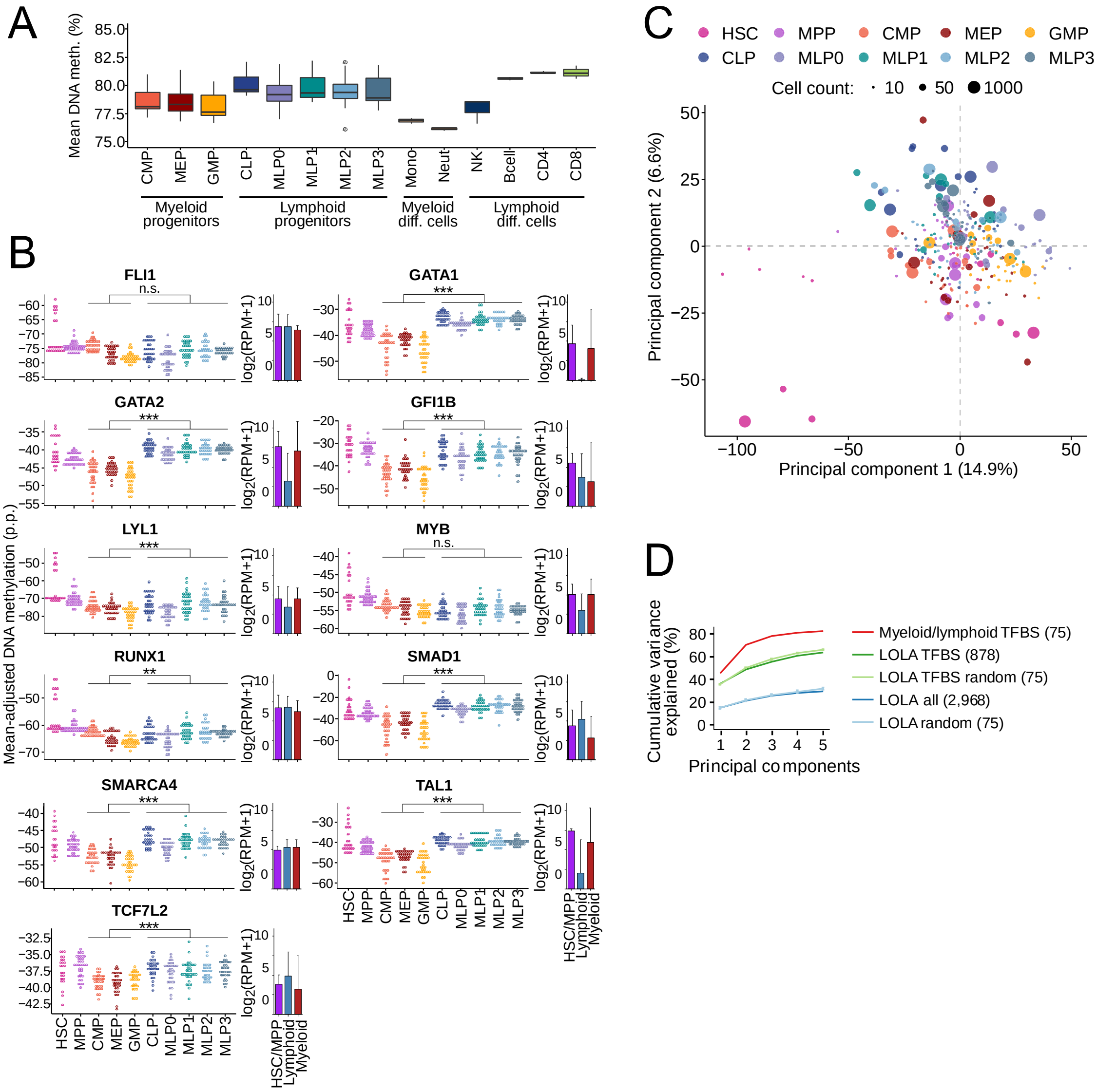

Figure S3. DNA methylation differences between myeloid and lymphoid progenitors

A) Distribution of average DNA methylation levels across BLUEPRINT Regulatory Build regions in progenitors and differentiated cell types of the myeloid and lymphoid lineages.

B) Mean-adjusted DNA methylation relative to the average CpG methylation levels for each individual 10-cell, 50-cell, and 1,000-cell sample averaged across ChIP-seq peaks for all enriched transcription factors shown in **Figure 3C**. The bar plots on the right of each diagram show the average gene expression levels of the corresponding transcription factors in HSCs/MPPs, in lymphoid progenitors (CLP, MLP0, MLP1, MLP2, MLP3), and in myeloid progenitors (CMP, MEP, GMP). Error bars correspond to the standard error. Brackets indicate two-tailed Wilcoxon tests with FDR-adjusted p-values. ***: $p \leq 0.001$, **: $p \leq 0.01$, n.s.: $p \geq 0.05$, p.p.: percentage points.

C) Two-dimensional projection of all 10-cell, 50-cell, and 1,000-cell samples from peripheral blood using principal component analysis based on the mean-adjusted DNA methylation across all 2,968 ChIP-seq region sets in the LOLA Core database. The first two principal components are shown, and the numbers in parentheses indicate the percentage of variance explained.

D) Cumulative percentage of variance explained by the first five principal components calculated from the mean-adjusted DNA methylation across all regions in the LOLA Core database (blue line), across 75 randomly selected datasets from this database averaged over 100 random samplings (light blue line), across all transcription factor binding sites (TFBS) from ENCODE and CODEX (green line), across 75 randomly selected datasets from these databases averaged over 100 random samplings (light green line), or across 75 transcription factor binding sites relevant to myeloid/lymphoid differentiation (red line) as in **Figure 3C**.

Related to Figure 3.

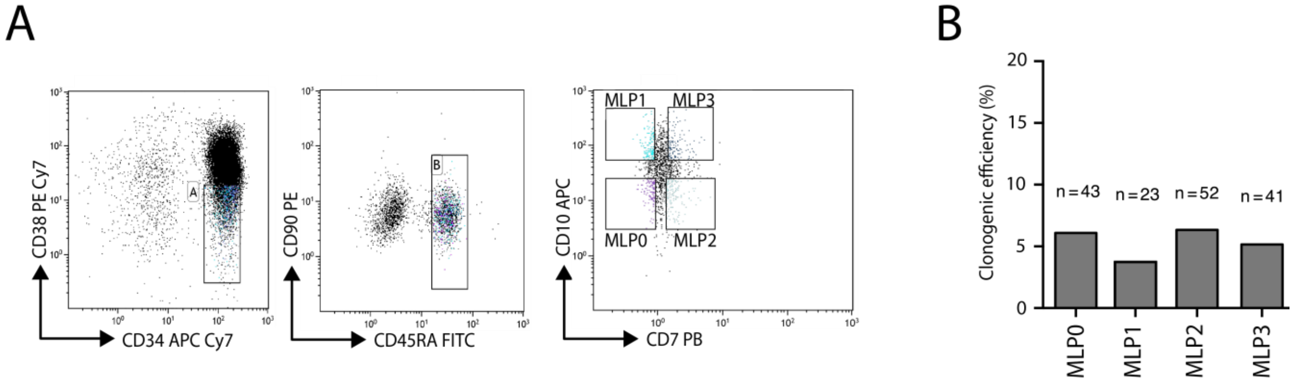

Figure S4. Sorting and *in vitro* differentiation of immature multi-lymphoid progenitors

A) Immature multi-lymphoid progenitor cells (MLP0, MLP1, MLP2, MLP3) were sorted from the CD34+, CD45RA+ fraction of peripheral blood based on the expression of CD10 and CD7.

B) Bar plots summarizing the clonogenic efficiency determined by *in vitro* colony formation assays for the four MLP populations. The total number of tested cells of each type is indicated.

Related to Figure 4.

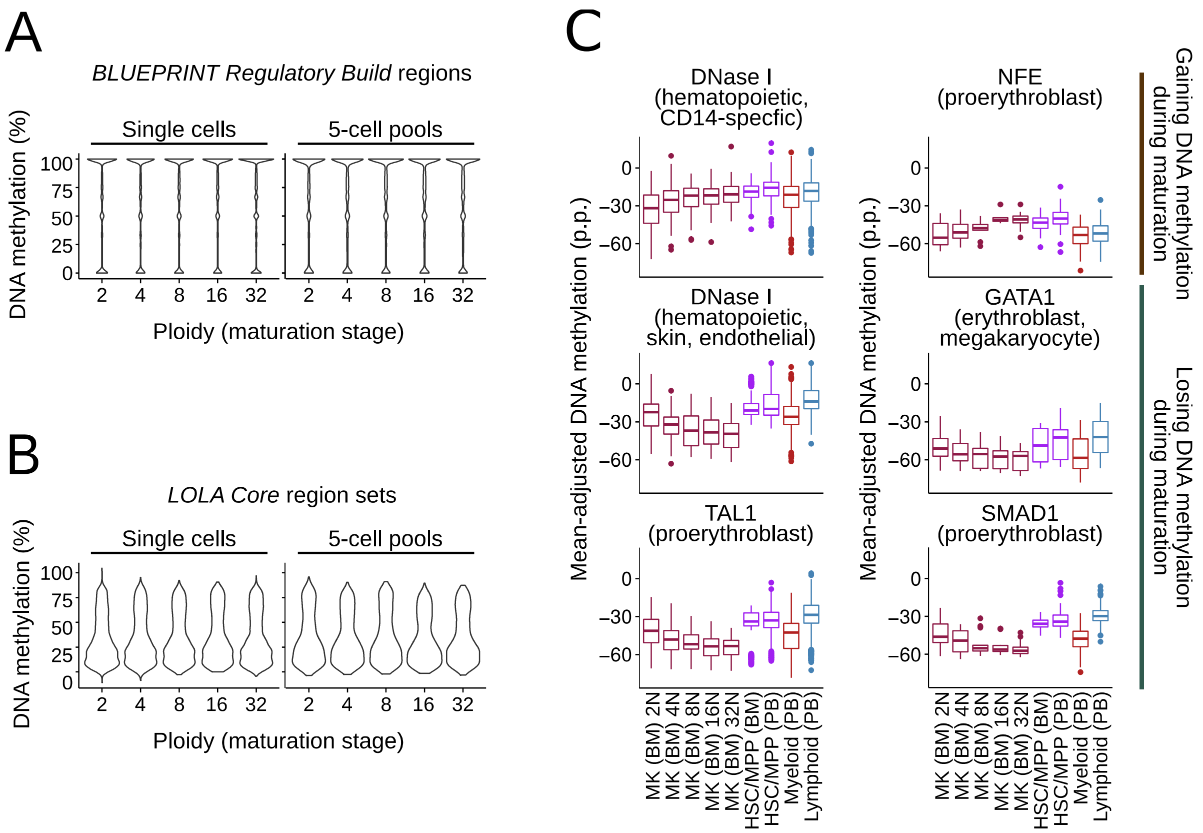

Figure S5. DNA methylation differences between megakaryocytes at different stages of ploidy

A) Violin plots showing the distribution of DNA methylation levels at BLUEPRINT Regulatory Build regions in megakaryocytes sorted according to their ploidy level (x-axis).

B) Violin plots showing the distribution of DNA methylation levels averaged across region sets in the LOLA Core database in megakaryocytes sorted according to their ploidy level (x-axis).

C) Distribution of mean-adjusted DNA methylation (relative to the average CpG methylation in each sample) across the region sets shown in **Figure 5C**. Megakaryocytes (MK) at different ploidy stages are compared to HSCs and MPPs sorted from bone marrow (BM) and peripheral blood (PB), and to myeloid progenitors (CMP, MEP, GMP) as well as lymphoid progenitors (CLP, MLP0, MLP1, MLP2, MLP3) sorted from peripheral blood.

Related to Figure 5.

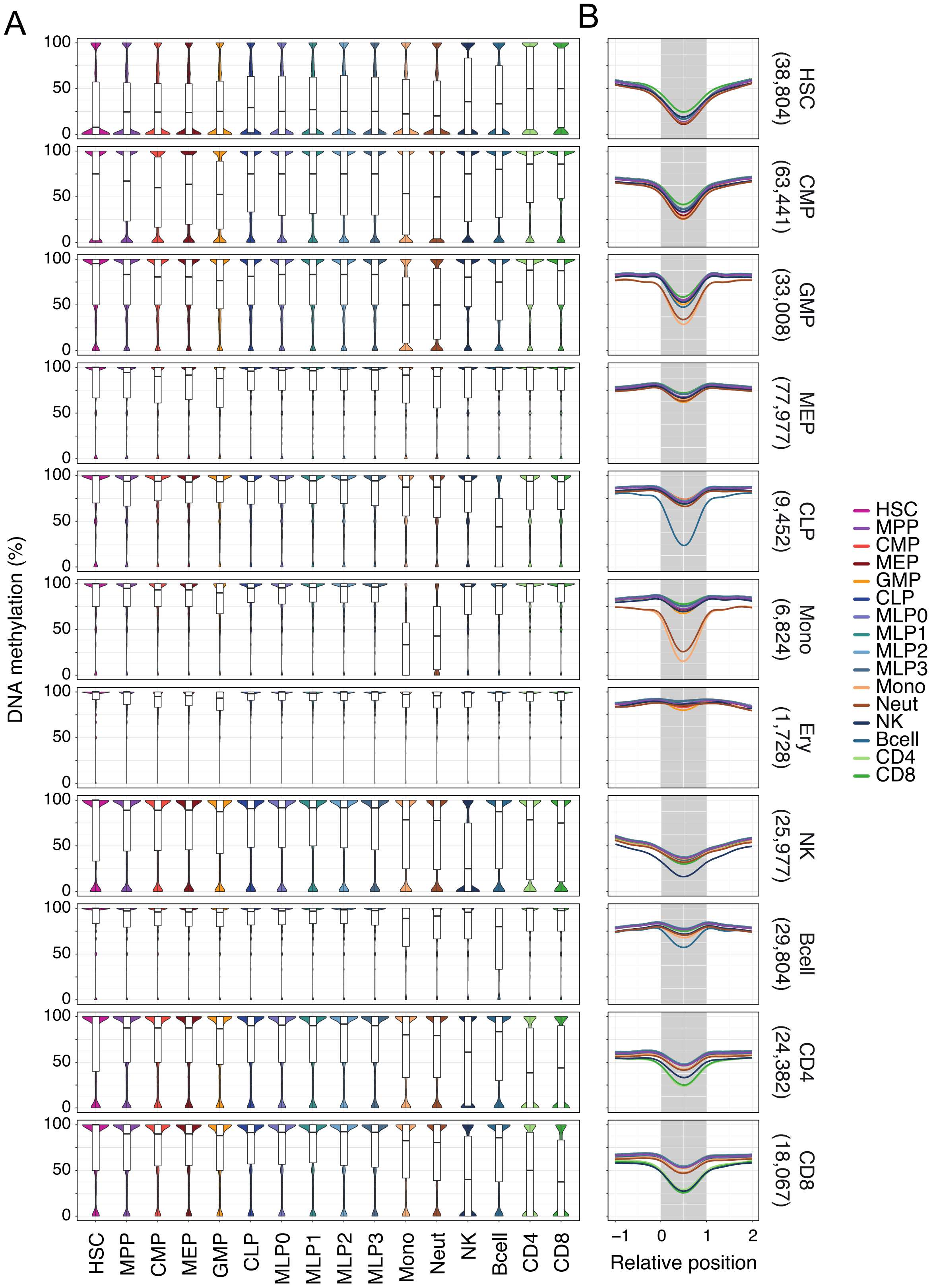

Figure S6. DNA methylation in regions with cell-type-specific chromatin accessibility

A) Boxplots showing the distribution of DNA methylation levels in regions with cell-type-specific chromatin accessibility based on published ATAC-seq data for hematopoietic cell types (GEO accession: GSE74912). The panel is an extended version of **Figure 6F**.

B) Composite plots showing DNA methylation averages across regions with cell-type-specific chromatin accessibility (numbers in parentheses). The panel is an extended version of **Figure 6G**.

Related to Figure 6.

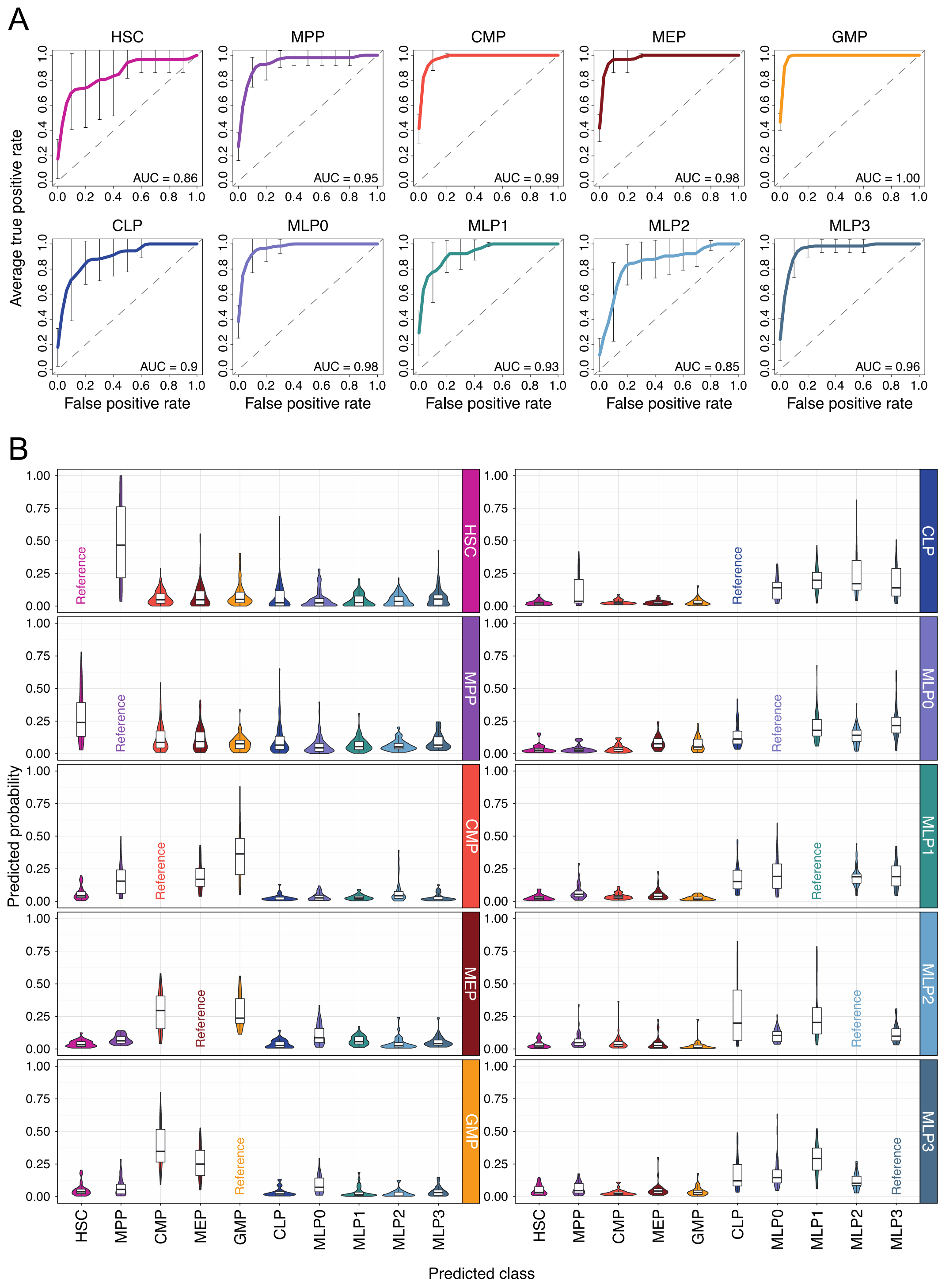

Figure S7. Prediction performance and class probability distributions for cell type classifiers

A) Receiver operating characteristic (ROC) curves and area under curve (AUC) values summarizing the prediction performance of elastic net-regularized general linear models that predict cell type from DNA methylation levels at BLUEPRINT Regulatory Build regions. The ROC curves plot the average true positive rate across 10-fold cross-validation against the false positive rate. They are based on one-versus-all prediction for each class, sliding a threshold along a value calculated as the difference of the class probability and the largest class probability excluding that class. Error bars correspond to standard deviations across the 10-fold cross-validation. Diagonal dashed lines indicate the expected performance of random guessing (AUC = 0.5).

B) Distribution of class probabilities by ten classifiers (shown in separate plots) trained on datasets that excluded all samples of one specific cell type (“leave-one-class-out classifiers”).

Related to Figure 7.

Supplemental Tables

Table S1. Sample annotations and sequencing statistics

Table listing the annotation data and sequencing details for 639 DNA methylation profiles based on the µWGBS / scWGBS protocol and for 13 gene expression profiles based on the Smart-seq2 protocol.

Related to Figure 1.

Table S2. Differentially methylated regions between hematopoietic cell types and lineages

Table listing all regulatory regions from the BLUEPRINT Regulatory Build that were differentially methylated in at least one pairwise comparison between HSCs and MPPs derived from four different sources or between the myeloid and lymphoid lineages in peripheral blood. An extended version of this table with additional comparisons is available from http://blueprint-methylomes.computational-epigenetics.org.

Related to Figures 2 and 3.

Table S3. Enrichment analysis for differentially methylated regions and cell type signature regions

Region set enrichment analysis for differentially methylated regions (**Table S2**) and cell type signature regions (**Table S4**) calculated using the LOLA software tool and the LOLA Core database.

Related to Figures 2, 3, and 7.

Table S4. Signature regions identified by the cell type classifier

Table listing all regulatory regions from the BLUEPRINT Regulatory Build that contributed to the cell type classifier trained on 319 stem and progenitor samples (all 10-cell, 50-cell, and 1,000-cell pools) from peripheral blood, together with the average DNA methylation level of each region in each sample.

Related to Figure 7.

Table S5. Classifier-based similarity among the stem and progenitor cell types

Class probabilities for each stem and progenitor sample based on ten classifiers trained on datasets that excluded all samples of one specific cell type ("leave-one-class-out classifiers").

Related to Figure 7.

Supplemental Experimental Procedures

Sample collection

Peripheral blood cells were isolated from apheresis filters of healthy platelet donors belonging to the NIHR Cambridge BioResource at the NHS Blood and Transplant, Cambridge, after informed consent and with ethical approval (REC 12/EE/0040). Bone marrow for megakaryocyte sorting was obtained from otherwise healthy patients undergoing elective heart valve replacement at Barts Health NHS Trust, London, after informed consent and with ethical approval (REC 13/LO/1760). Bone-marrow-derived CD34+ cells for HSC/MPP sorting were purchased from Lonza, cat. 2M-101D (lots 0000536591, 0000476376 and 0000536050). Cord blood was collected at the Rosie Maternity Hospital, Cambridge University Hospitals, after informed consent and with ethical approval (REC 12/EE/0040). Fetal liver-derived CD34+ cells were purchased from StemExpress, cat. FL0001C (lots 1508211059, 405585112, and 1602050036).

Cell purification overview

Peripheral blood cells were extracted from apheresis filters and layered on a Ficoll-Paque gradient to isolate the fraction of mononuclear cells. After washing, the cells were processed by autoMACS (Miltenyi) to enrich for the CD34+ fraction using the posseld2 program. Cells were then stained with antibodies described below for 45 minutes at 4°C and subsequently sorted on either BD Influx or BD FACS Aria III fluorescence-activated cell sorting instruments. Bone-marrow-derived and fetal liver-derived CD34+ cells were thawed in a water bath at 37°C and resuspended in PBS1x plus DNase (10 mg/ml). After washing, the cells were stained as described above. Megakaryocytes were isolated from bone marrow as follows: A bone marrow scraping was taken after median sternotomy using a Volkmann's spoon. The sample was transported to the University of Cambridge for processing as whole bone marrow in phosphate buffered saline (PBS) containing 10% human serum albumin (HSA) and 1.8 mg/ml EDTA on ice. The cellular content was flushed out of the bone marrow using megakaryocyte buffer (PBS containing 1.2% HSA, 1.8 mg/ml EDTA), and red cells were lysed using ammonium chloride lysis. The cells were stained for megakaryocyte-specific cell surface markers with mouse APC conjugated antibody against CD41a (BD), mouse PE conjugated antibody against CD42b (BD), and for ploidy analysis with 1ug/ml Hoechst 33342 (Invitrogen). After incubation at 37°C for 30 minutes the cells were kept at 4°C before sorting using a BD FACS Aria instrument.

The cell populations were sorted using the following surface markers: HSC: Lin- CD34+ CD38- CD90+ CD45RA- CD49f+; MPP: Lin- CD34+ CD38- CD90- CD45RA- CD49f-; CMP: Lin- CD34+ CD38+ CD45RA- CD123 low; MEP: Lin- CD34+ CD38+ CD45RA- CD123- FLT3- CD36- CD110+ CD41-; GMP: Lin- CD34+ CD38+ CD45RA+ CD123+ CD10-; CLP: Lin- CD34+ CD38+ CD45RA+ CD7- CD10+; MLP0: Lin- CD34+ CD38- CD90- CD45RA+ CD7- CD10-; MLP1: Lin- CD34+ CD38- CD90- CD45RA+ CD7- CD10+; MLP2: Lin- CD34+ CD38- CD90- CD45RA+ CD7+ CD10-; MLP3: Lin- CD34+ CD38- CD90- CD45RA+ CD7+ CD10+; Megakaryocyte: CD41a+ CD42b+ Hoechst; Monocyte: CD14+ CD16- CD45+ CD64+; Neutrophil: CD16+ CD45+ CD66b+; NK cell: CD3- CD16+ CD56 low; B cell (naïve): Cd19+ CD27- IgD+; CD4 T cell (naïve): CD3+ CD4+ CD25- CD45RA+ CD62l+; CD8 T cell (naïve): CD3+ CD8+ CD25- CD45RA+ CD62L+.

Cell purification details

Isolation of CD34+ cells from apheresis filters

- Remove blood from filter into 50 ml falcon tube
- Dilute the blood up to 50 ml with room temperature Buffer 1
- Add 12.5 ml of Ficoll-Paque to two 50 ml falcon tubes
- Carefully pipette 25 ml of cell suspension on the Ficoll
- Spin 15 minutes, 800 g ↑3 ↓0
- Carefully remove the mononuclear cell layer using a 5 ml pastette
- Transfer the mononuclear cells into a fresh 50 ml tube
- Fill the tubes to 50 ml with Buffer 1 (to remove more platelets)
- Spin 6 minutes, 600 g ↑5 ↓3 (cold)
- Remove the supernatant
- Pool tubes into one 50 ml falcon tube and re-suspend in a total of 50 ml of cold Buffer 4

- Count the cells
- Spin for 6 minutes at 600 g ↑5 ↓3 (4°C)
- Remove the supernatant

Magnetic labelling and CD34+ enrichment

- Re-suspend pellet in 150µl of Buffer 4 per 10^8 cells (e.g., $9.6x10^8$ 1440 µl)
- Add 50 µl of FcR blocking reagent per 10^8 cells (e.g., $9.6x10^8$ 480 µl)
- Add 50 µl of CD34 microbeads per 10^8 cells
- Put the cells in 4°C for 30 minutes
- Add 20 ml of Buffer 4
- Spin for 6 minutes at 300 g ↑5 ↓3
- Remove supernatant
- Re-suspend pellet in 500 µl of Buffer 4 per 10^8 cells (e.g., $9.6x10^8$ 4.8 ml)
- Run sample on autoMACS using program posseld2
- Count the cells
- Stain with 1 test per 10^6 cells

Materials

- Ficoll-Paque density 1.077 (GE Healthcare, cat. 17-5442-03)
- CD34 MicroBead Kit human (Miltenyi Biotec, cat. 130-046-703) 10 ml
- PBS (Sigma, cat. D8537) 500 ml
- HAS (Gemini Bio Products, cat. 800-121)
- EDTA (Sigma, cat. E7889) 50 ml
- BSA (Sigma, cat. A9576)

Buffer 1

- PBS (Sigma, cat. D8537) 500 ml
- 1 M TriSodium Citrate 6.6 ml
- HSA 20% (0.2% final) 5 ml

Buffer 4

- PBS (Sigma, cat. D8537) 500 ml
- 0.5 M EDTA (Sigma, cat. E7889 50 ml) 2 ml (2 mM final)
- HSA 20% (0.2% final) 5 ml

Cell purification antibodies

Conjugate	Antigen	Name	Manufacturer	Product number	Concentration
**AF700**	CD3	OKT3	BioLegend	317339	5 µl/test
**AF700**	CD56	B159	BD Biosciences	557919	5 µl/test
**AF700**	CD8	SK1	BioLegend	344723	5 µl/test
**AF700**	CD14	61D3	BD Biosciences	56-0149-42	5 µl/test
**AF700**	CD11B	CBRM1/5	BD Biosciences	56-0113-42	5 µl/test
**AF700**	CD19	H1B19	BioLegend	302225	5 µl/test
**PE**	CD90	5E10	BD Biosciences	561970	5 µl/test
**PE CY 5**	CD49F	G0H3	BD Biosciences	551129	20 µl/test
**APC CY 7**	CD34	581	Molecular Probes	A14948	5 µl/test
**APC**	CD10	HI10A	BD Biosciences	332777	5 µl/test
**FITC**	CD45RA	L48	BD Biosciences	335039	20 µl/test
**PE CY 7**	CD38	HB7	BD Biosciences	335825	5 µl/test
**PB**	CD7	MT701	BD Biosciences	642916	20 µl/test
**PerCP-Cy 5.5**	CD123	7G3	BD Biosciences	560904	20 µl/test

Clonal expansion assays

Sorted single cells of the CLP, MLP0, MLP1, MLP2, and MLP3 cell populations were cultured on MS5 stroma (Itoh et al., 1989) for three weeks in conditions that support myeloid, B cell, and NK cell differentiation (Laurenti et al., 2013). Colonies were harvested, and differentiated cell types were scored by high-throughput flow cy-

tometry using the LSR II High Throughput Sampler (Becton Dickinson) with the following antibodies (Biolegend): CD45 PECy5 (1:300), CD41 FITC (1:1000), GlyA PE (BD, 1:1000), CD11b APCCy7 (1:300), CD56 APC (1:200), CD19 FITC (1:200), CD19 Alexa700 (1:300).

Whole genome bisulfite sequencing

Sequencing libraries for DNA methylation mapping were prepared using the µWGBS protocol (Farlik et al., 2015). Starting directly from lysed cells in digestion buffer, proteinase K digestion was performed at 50°C for 20 minutes. Custom-designed methylated and unmethylated oligonucleotides were added at a concentration of 0.1% to serve as spike-in controls for monitoring bisulfite conversion efficiency. Bisulfite conversion was performed using the EZ DNA Methylation-Direct Kit (Zymo Research, D5020) according to the manufacturer's protocol, with the modification of eluting the DNA in only 9 µl of elution buffer. Bisulfite-converted DNA was used for single-stranded library preparation using the EpiGnome Methyl-Seq kit (Epicentre, EGMK81312) with the described modifications (Farlik et al., 2015). Quality control of the final library was performed by measuring DNA concentrations using the Qubit dsDNA HS assay (Life Technologies, Q32851) on Qubit 2.0 Fluorometer (Life Technologies, Q32866) and by determining library fragment sizes with the Agilent High Sensitivity DNA Analysis kit (Agilent, 5067-4626) on Agilent 2100 Bioanalyzer Station (Agilent, G2939AA). All libraries were sequenced by the Biomedical Sequencing Facility at CeMM using the 2x75bp paired-end setup on the Illumina HiSeq 3000/4000 platform (see **Table S1** for sequencing statistics).

DNA methylation data processing

Sequencing adapter fragments were trimmed using Trimmomatic v0.32 (Bolger et al., 2014). The trimmed reads were aligned with Bismark v0.12.2 (Krueger and Andrews, 2011) with the following parameters: *--minins 0 --maxins 6000 --bowtie2*, which uses Bowtie2 v2.2.4 (Langmead and Salzberg, 2012) for read alignment. The GRCh38 assembly of the human reference genome was used throughout the study, in a version for sequence alignment obtained from NCBI. Duplicate reads were removed as potential PCR artefacts, and reads with a bisulfite conversion rate below 90% or with fewer than three cytosines outside a CpG context (required to confidently assess bisulfite conversion rate) were removed as potential post-bisulfite contamination. The Bismark extractor was used to estimate DNA methylation levels for each CpG. Replicates belonging to the same individual and cell type were merged by summing up the total number of methylated and unmethylated reads per CpG across all replicates. Merged and unmerged datasets were processed further using RnBeads v1.5 (Assenov et al., 2014) to generate standard analysis reports for data exploration and quality control (http://blueprint-methylomes.computational-epigenetics.org), and to aggregate DNA methylation values of individual CpGs based on genomic tiling regions (width: 5 kilobases) and based on regulatory regions annotated by the August 2015 release of the BLUEPRINT Ensembl Regulatory Build (Zerbino et al., 2015). The DNA methylation tables produced by RnBeads were the basis for further data analysis with custom R scripts.

Differential DNA methylation analysis

We analyzed differential DNA methylation for regulatory regions defined by the August 2015 release of the BLUEPRINT Ensembl Regulatory Build (**Figure 2B, 3A, Table S2**). All pairwise comparisons were performed with the differential methylation module in RnBeads (Assenov et al., 2014), which uses the limma method for statistical analysis (Ritchie et al., 2015). Potential confounding factors such as flowcell, gender, and number of cells sequenced were statistically accounted for in the RnBeads analysis. Regions were considered differentially methylated if they had an FDR-adjusted p-value below 0.05 and an absolute change in DNA methylation that was among the top 5% strongest absolute differences observed across all pairwise comparisons (which corresponds to a difference in absolute DNA methylation levels of at least 16.7 percentage points). We further removed regions that had not been covered in at least three samples and regions that had not been covered with at least three reads in at least one sample.

Region set enrichment analysis

We used LOLA (Sheffield and Bock, 2015) to identify significant overlaps of differentially methylated regions and cell type signature regions with empirically defined transcription factor binding sites based on ChIP-seq datasets obtained from ENCODE (Harrow et al., 2012) and from the CODEX database (Sánchez-Castillo et al., 2015). Fisher's exact test was used with a significance threshold of 0.05 on Benjamini-Yekutieli adjusted p-values. Figure panels include all transcription factors that were enriched in at least one of the relevant comparisons, while also showing enrichment data for these transcription factors in cell types where they were not enriched. To facilitate visualization and interpretation, we manually grouped the annotations into broader categories. All enriched results together with their original and curated annotations are available in **Table S3**. ChIP-seq datasets for malignant cell populations were excluded from the figures given the study's focus on normal hematopoietic differentiation (but they are included in **Table S3**).

Single-cell DNA methylation analysis

To compensate for the sparseness of low-input and single-cell DNA methylation data, several analyses (**Figure 3D-G**, **S3B-D**, **4B**, **4C**, **5B**, **5C**, **S5B**, **S5C**) employed a region set analysis strategy described previously (Farlik et al, 2015). This bioinformatic method is based on the observation that characteristic cell-type-specific DNA methylation differences can be identified by calculating average DNA methylation levels across sets of functionally related regions (e.g., across binding sites of a transcription factor or enhancer elements active in a certain cell type). We used the LOLA Core database (Sheffield & Bock, 2015), a large catalog of experimentally identified regulatory region sets, as our reference. For each stem and progenitor dataset we calculated average DNA methylation levels across each region set. We adjusted these values for differences in global DNA methylation levels between cell types by subtracting, in each sample, the global DNA methylation average across all CpGs from the region set values. Analyses based on individual low-input and single-cell samples used these region set estimates of mean-adjusted DNA methylation (relative to the average CpG methylation level in each sample) in the same way as analyses based on pooled replicates used region-level DNA methylation data for the BLUEPRINT Regulatory Build. We used Wilcoxon rank sum tests and considered region sets with a p-value ≤ 0.05 and an absolute change in mean-adjusted DNA methylation of at least 10 percentage points as differentially methylated.

RNA sequencing

Cells were sorted directly into lysis buffer containing 0.2% Triton X-100 and RNase inhibitor. The cDNA synthesis and poly(A) enrichment were performed following the Smart-seq2 protocol (Picelli et al., 2014). ERCC spike-in RNA (Ambion) was added to the lysis buffer in a dilution of 1:1,000,000. Library preparation was performed on 0.5 ng cDNA using the Nextera XT library preparation kit (Illumina) following the manufacturer instructions. All libraries were sequenced by the Biomedical Sequencing Facility at CeMM using the 1x50 bp single-read setup on the Illumina HiSeq 3000/4000 platform (see **Table S1** for sequencing statistics).

Gene expression analysis

Sequencing adapter fragments were trimmed using Trimmomatic v0.32 (Bolger et al., 2014). The trimmed reads were aligned to the cDNA reference transcriptome (GRCh38 cDNA sequences from Ensembl) using Bowtie v1.1.1 (Langmead et al., 2009) and the following parameters: *-q -p 6 -a -m 100 --minins 0 --maxins 5000 --fr --sam --chunkmbs 200*. Duplicate reads were removed, and transcript levels were quantified with BitSeq v1.12.0 (Glaus et al., 2012). Transcript-level expression estimates were loaded into R and collapsed into gene-level estimates by using the most highly expressed transcript variant. DESeq2 (Love et al., 2014) was used for statistical analysis of the read counts. Genes with an FDR-corrected p-value ≤ 0.05 and at least a two-fold change in expression ($|\log_2 FC| \geq 1$) were considered as differentially expressed. Gene expression estimates for visualization and reporting were adjusted by variance stabilization.

Integration of histone modification data

We processed all histone data of the September 2015 BLUEPRINT release (seventh data release) using a similar approach as in the Roadmap Epigenomics analysis (Ernst and Kellis, 2015; Kundaje et al., 2015). Briefly, we selected all samples for which the input control and at least three of the six histone modifications (H3K27ac, H3K27me3, H3K36me3, H3K4me1, H3K4me3, H3K9me3) were available and generated genome-wide tracks for the ChIP-seq signal enrichment over input using MACS2 v2.1.0 (Zhang et al., 2008). These tracks were used as input to ChromImpute v1.0.0 (Ernst and Kellis, 2015), imputing all missing data and merging replicates. The p-values calculated by MACS2 were used as intensity estimates for the boxplots.

Integration of open chromatin data

We downloaded peak regions and fragment counts from ATAC-seq experiments (GEO accession GSE74912) for hematopoietic cell types (Corces et al., 2016) and transformed the peak coordinates to genome assembly GRCh38 using the UCSC liftOver tool (https://genome.ucsc.edu/cgi-bin/hgLiftOver). Average DNA methylation levels across samples were computed for all ATAC-seq peaks. We used the one-sided Wilcoxon rank sum test to identify cell-type-specific regions of open chromatin. Specifically, for each cell type in the ATAC-seq data set we selected those peak regions in which samples of that cell type exhibited a significantly higher ATAC-seq fragment count than samples not belonging to that cell types (FDR adjusted p-value ≤ 0.05).

Cell type prediction

Samples were classified using elastic net-regularized general linear models as implemented in the R package *glmnet* (Friedman et al., 2010; Krishnapuram et al., 2005). Classifiers were trained on 319 stem and progenitor samples (all 10-cell, 50-cell, and 1,000-cell pools) from peripheral blood, using their DNA methylation profiles across regulatory regions from the BLUEPRINT Ensembl Regulatory Build as prediction variables. Missing values were imputed with the *impute* R package (https://bioconductor.org/packages/release/bioc/html/impute.html) using 5-nearest neighbor averaging. Elastic net regularization was applied to a multinomial logistic regression classifier. The regularization parameter λ was obtained by nested 10-fold cross-validation, and α was set to 0.5 to stipulate equal mixing of the lasso and ridge penalty terms. Class importance was defined as the L2 norm aggregate of per-class coefficients in the model. Class probabilities were defined as fitted probabilities from the logistic regression model. For assessing model quality, 10-fold cross validation was performed and misclassification rates were averaged over the cross-validation test sets. Per-class ROC curves and area under curve (AUC) values were obtained by evaluating the class probabilities in the one-versus-all setting for each class, i.e., by sliding a threshold along the score resulting from the difference of the class probability and the largest class probability excluding that class. Signature regions were defined as those regulatory regions that were assigned a non-zero class importance value in the model trained on the entire dataset (**Table S4**). For quantifying class probabilities of individual cell types (**Figures S7B, Table S5**), the samples of one class were excluded from the training, and the resulting model applied to the samples excluded from training ("leave-one-class-out classifiers").

Inference of cell type similarity graph

In the cell type similarity graph (**Figure 7F**), nodes represent cell types and edges represent probabilities of predicting one cell type as another using the corresponding "leave-one-class-out" classifier. Specifically, for each pair of source and target cell type, the edge weight is the average class probability assigned by the leave-one-class-out classifier to all peripheral blood samples of the source cell type to the target cell type. The graph shows the directed edge pairs for each pair of nodes as trapezoids in which the widths at the target and source node correspond to weights of the directed edges. For example, the predictor that did not include HSCs assigned a higher probability to classify HSCs as MPPs than the probabilities the predictor which did not include MPPs assigned to predicting MPPs as HSCs. Differentiated cell types (circles) were predicted based on the classifier trained on all 319 stem and progenitor samples from peripheral blood and are connected by grey edges in the graph. Edges with a prediction probability below 0.1 were pruned. The graph layout was automatically generated using the Fruchterman-Reingold algorithm as implemented in the R package *igraph*.

Supplemental References

Assenov, Y., Müller, F., Lutsik, P., Walter, J., Lengauer, T., and Bock, C. (2014). Comprehensive analysis of DNA methylation data with RnBeads. Nat Methods *11*, 1138-1140.

Bolger, A.M., Lohse, M., and Usadel, B. (2014). Trimmomatic: a flexible trimmer for Illumina sequence data. Bioinformatics *30*, 2114–2120.

Corces, M.R., Buenrostro, J.D., Wu, B., Greenside, P.G., Chan, S.M., Koenig, J.L., Snyder, M.P., Pritchard, J.K., Kundaje, A., Greenleaf, W.J., *et al.* (2016). Lineage-specific and single-cell chromatin accessibility charts human hematopoiesis and leukemia evolution. Nat Genet 48, 1193–1203.

Ernst, J., and Kellis, M. (2015). Large-scale imputation of epigenomic datasets for systematic annotation of diverse human tissues. Nat Biotechnol *33*, 364–376.

Farlik, M., Sheffield, N.C., Nuzzo, A., Datlinger, P., Schönegger, A., Klughammer, J., and Bock, C. (2015). Single-cell DNA methylome sequencing and bioinformatic inference of epigenomic cell-state dynamics. Cell Rep *10*, 1386-1397.

Friedman, J., Hastie, T., and Tibshirani, R. (2010). Regularization Paths for Generalized Linear Models via Coordinate Descent. J Stat Softw *33*, 1–22.

Glaus, P., Honkela, A., and Rattray, M. (2012). Identifying differentially expressed transcripts from RNA-seq data with biological variation. Bioinformatics *28*, 1721-1728.

Harrow, J., Frankish, A., Gonzalez, J.M., Tapanari, E., Diekhans, M., Kokocinski, F., Aken, B.L., Barrell, D., Zadissa, A., Searle, S., et al. (2012). GENCODE: The reference human genome annotation for The ENCODE Project. Genome Res *22*, 1760–1774.

Itoh, K., Tezuka, H., Sakoda, H., Konno, M., Nagata, K., Uchiyama, T., Uchino, H., and Mori, K.J. (1989). Reproducible establishment of hemopoietic supportive stromal cell lines from murine bone marrow. Exp Hematol *17*, 145-153.

Krishnapuram, B., Carin, L., Figueiredo, M.A.T., and Hartemink, A.J. (2005). Sparse multinomial logistic regression: fast algorithms and generalization bounds. IEEE Trans Pattern Anal Mach Intell *27*, 957–968.

Krueger, F., and Andrews, S.R. (2011). Bismark: a flexible aligner and methylation caller for bisulfite-seq applications. Bioinformatics *27*, 1571-1572.

Kundaje, A., Meuleman, W., Ernst, J., Bilenky, M., Yen, A., Heravi-Moussavi, A., Kheradpour, P., Zhang, Z., Wang, J., et al. (2015). Integrative analysis of 111 reference human epigenomes. Nature *518*, 317–330.

Langmead, B. and Salzberg, S.L. (2012). Fast gapped-read alignment with Bowtie 2. Nat Methods *9*, 357-359.

Langmead, B., Trapnell, C., Pop, M., and Salzberg, S.L. (2009). Ultrafast and memory-efficient alignment of short DNA sequences to the human genome. Genome Biol *10*, R25.

Laurenti, E., Doulatov, S., Zandi, S., Plumb, I., Chen, J., April, C., Fan, J.B., and Dick, J.E. (2013). The transcriptional architecture of early human hematopoiesis identifies multilevel control of lymphoid commitment. Nat Immunol *14*, 756-763.

Love, M.I., Huber, W., and Anders, S. (2014). Moderated estimation of fold change and dispersion for RNA-seq data with DESeq2. Genome Biol *15*, 550.

Picelli, S., Faridani, O.R., Bjorklund, A.K., Winberg, G., Sagasser, S., and Sandberg, R. (2014). Full-length RNA-seq from single cells using Smart-seq2. Nat Protoc *9*, 171-181.

Ritchie, M.E., Phipson, B., Wu, D., Hu, Y., Law, C.W., Shi, W., and Smyth, G.K. (2015). limma powers differential expression analyses for RNA-sequencing and microarray studies. Nucleic Acids Res *43*, e47.

Sánchez-Castillo, M., Ruau, D., Wilkinson, A.C., Ng, F.S.L., Hannah, R., Diamanti, E., Lombard, P., Wilson, N.K., and Göttgens, B. (2015). CODEX: a next-generation sequencing experiment database for the haematopoietic and embryonic stem cell communities. Nucleic Acids Res. *43*, D1117–D1123.

Sheffield, N.C., and Bock, C. (2016). LOLA: enrichment analysis for genomic region sets and regulatory elements in R and Bioconductor. Bioinformatics *32*, 587-589.

Zerbino, D.R., Wilder, S.P., Johnson, N., Juettemann, T., and Flicek, P.R. (2015). The Ensembl Regulatory Build. Genome Biol. *16*, 56.

Zhang, Y., Liu, T., Meyer, C.A., Eeckhoute, J., Johnson, D.S., Bernstein, B.E., Nusbaum, C., Myers, R.M., Brown, M., Li, W., et al. (2008). Model-based analysis of ChIP-Seq (MACS). Genome Biol. *9*, R137.
