# Supplementary material for: DNA Methylation Dynamics of Human Hematopoietic Stem Cell Differentiation
Source: Cell Stem Cell. 2016 Dec 1;19(6):808–22. doi: 10.1016/j.stem.2016.10.019 (PMC5145815; doi:10.1016/j.stem.2016.10.019)
Supplement: Document S2. Article plus Supplemental Information [file mmc7.pdf]

Resource

Cell Stem Cell

DNA Methylation Dynamics of Human Hematopoietic Stem Cell Differentiation

Graphical Abstract

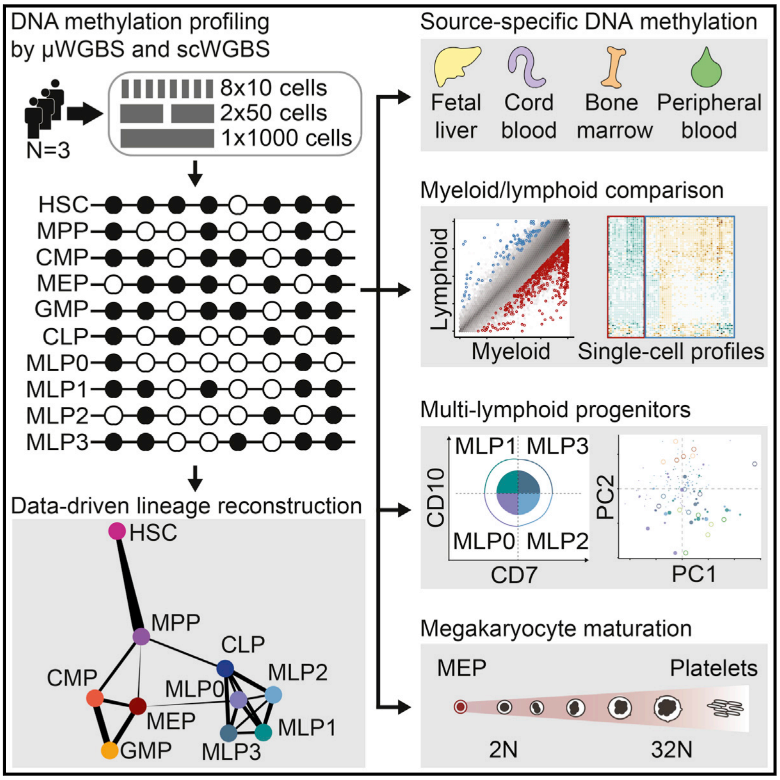

Highlights

- Sequencing provides DNA methylation maps of hematopoietic stem and progenitor cells
- Methylation differs in HSCs from fetal liver, bone marrow, cord, and peripheral blood
- Myeloid and lymphoid progenitors are distinguished by enhancer-linked DNA methylation
- Machine learning enables data-driven reconstruction of the hematopoietic lineage

Authors

Matthias Farlik, Florian Halbritter, Fabian Müller, ..., Thomas Lengauer, Mattia Frontini, Christoph Bock

Correspondence

mf471@cam.ac.uk (M.F.),
cbock@cemm.oeaw.ac.at (C.B.)

In Brief

As part of the IHEC consortium, Bock and colleagues present genome-wide reference maps of DNA methylation dynamics during human blood development. The characteristic DNA methylation patterns they see in the different cell types allow data-driven inference of an epigenome-based model of hematopoietic differentiation. Explore the IHEC web portal at http://www.cell.com/consortium/IHEC.

Farlik et al., 2016, Cell Stem Cell *19*, 808–822
December 1, 2016 © 2016 The Author(s). Published by Elsevier Inc.
http://dx.doi.org/10.1016/j.stem.2016.10.019

Cell Stem Cell
Resource

DNA Methylation Dynamics of Human Hematopoietic Stem Cell Differentiation

Matthias Farlik,[1,13] Florian Halbritter,[1,13] Fabian Müller,[2,3,13] Fizzah A. Choudry,[4,5] Peter Ebert,[2,3] Johanna Klughammer,[1] Samantha Farrow,[4,5] Antonella Santoro,[6] Valerio Ciaurro,[6] Anthony Mathur,[7] Rakesh Uppal,[7] Hendrik G. Stunnenberg,[8] Willem H. Ouwehand,[4,5,9,10] Elisa Laurenti,[4,6] Thomas Lengauer,[2] Mattia Frontini,[4,5,9,*] and Christoph Bock[1,2,11,12,14,*]
[1]CeMM Research Center for Molecular Medicine of the Austrian Academy of Sciences, 1090 Vienna, Austria
[2]Max Planck Institute for Informatics, Saarland Informatics Campus, 66123 Saarbrücken, Germany
[3]Graduate School of Computer Science, Saarland University, 66123 Saarbrücken, Germany
[4]Department of Haematology, University of Cambridge, Cambridge CB2 0PT, UK
[5]National Health Service Blood and Transplant, Cambridge Biomedical Campus, Cambridge CB2 0PT, UK
[6]Wellcome Trust-Medical Research Council Cambridge Stem Cell Institute, University of Cambridge, Clifford Allbutt Building, Hills Road, Cambridge CB2 0AH, UK
[7]Department of Cardiology, Barts Heart Centre, St Bartholomew's Hospital, Barts Health NHS Trust, London EC1A 7BE, UK
[8]Faculty of Science, Department of Molecular Biology, Radboud University, 6525GA Nijmegen, the Netherlands
[9]British Heart Foundation Centre of Excellence, Cambridge Biomedical Campus, Long Road, Cambridge CB2 0QQ, UK
[10]Department of Human Genetics, The Wellcome Trust Sanger Institute, Wellcome Trust Genome Campus, Hinxton, Cambridge CB10 1HH, UK
[11]Department of Laboratory Medicine, Medical University of Vienna, 1090 Vienna, Austria
[12]Ludwig Boltzmann Institute for Rare and Undiagnosed Diseases, 1090 Vienna, Austria
[13]Co-first author
[14]Lead Contact
*Correspondence: mf471@cam.ac.uk (M.F.), cbock@cemm.oeaw.ac.at (C.B.)
http://dx.doi.org/10.1016/j.stem.2016.10.019

SUMMARY

Hematopoietic stem cells give rise to all blood cells in a differentiation process that involves widespread epigenome remodeling. Here we present genome-wide reference maps of the associated DNA methylation dynamics. We used a meta-epigenomic approach that combines DNA methylation profiles across many small pools of cells and performed single-cell methylome sequencing to assess cell-to-cell heterogeneity. The resulting dataset identified characteristic differences between HSCs derived from fetal liver, cord blood, bone marrow, and peripheral blood. We also observed lineage-specific DNA methylation between myeloid and lymphoid progenitors, characterized immature multi-lymphoid progenitors, and detected progressive DNA methylation differences in maturing megakaryocytes. We linked these patterns to gene expression, histone modifications, and chromatin accessibility, and we used machine learning to derive a model of human hematopoietic differentiation directly from DNA methylation data. Our results contribute to a better understanding of human hematopoietic stem cell differentiation and provide a framework for studying blood-linked diseases.

INTRODUCTION

All blood cells originate from hematopoietic stem cells (HSCs), which represent the apex of a differentiation cascade of progenitor cell types that gives rise to billions of new cells every day. HSC differentiation is believed to progress through stepwise restriction of lineage potential, a concept that is summarized by the classical tree model of murine hematopoiesis (Spangrude, 1991; Till and McCulloch, 1980).

HSC differentiation in human is less well understood than in mouse. Despite recent progress (reviewed in Doulatov et al., 2012; Theocharides et al., 2016; Vedi et al., 2016), several aspects of human hematopoiesis have remained controversial (Chen et al., 2014; Doulatov et al., 2010; McCracken et al., 2013; Notta et al., 2016; Park et al., 2008; Tanner et al., 2014; Woolthuis and Park, 2016).

We sought to use DNA methylation for in vivo dissection of human hematopoiesis. DNA methylation is well suited for studying cellular differentiation because its patterns are cell-type-specific and retain an epigenetic memory of a cell's developmental history. For example, cell-of-origin-specific DNA methylation patterns are detectable among induced pluripotent stem cells (Kim et al., 2011; Polo et al., 2010), and such patterns of epigenetic tissue memory predict primary tumor location in metastatic cancers (Fernandez et al., 2012; Moran et al., 2016).

Previous studies have established a close connection between stem cell differentiation and widespread epigenome remodeling. DNA methylation has been studied in early mammalian development (Smallwood et al., 2011; Smith et al., 2012), mouse HSC differentiation (Bock et al., 2012; Cabezas-Wallscheid et al., 2014; Ji et al., 2010), neural differentiation (Lister et al., 2013), pluripotent stem cells (Bock et al., 2011; Habibi et al., 2013), and a broad collection of human tissue samples (Kundaje et al., 2015; Ziller et al., 2013). Chromatin accessibility has been mapped using the assay for transposase-accessible chromatin with high throughput sequencing (ATAC-seq) in multiple cell types of

 © 2016 The Author(s). Published by Elsevier Inc.
This is an open access article under the CC BY license (http://creativecommons.org/licenses/by/4.0/).

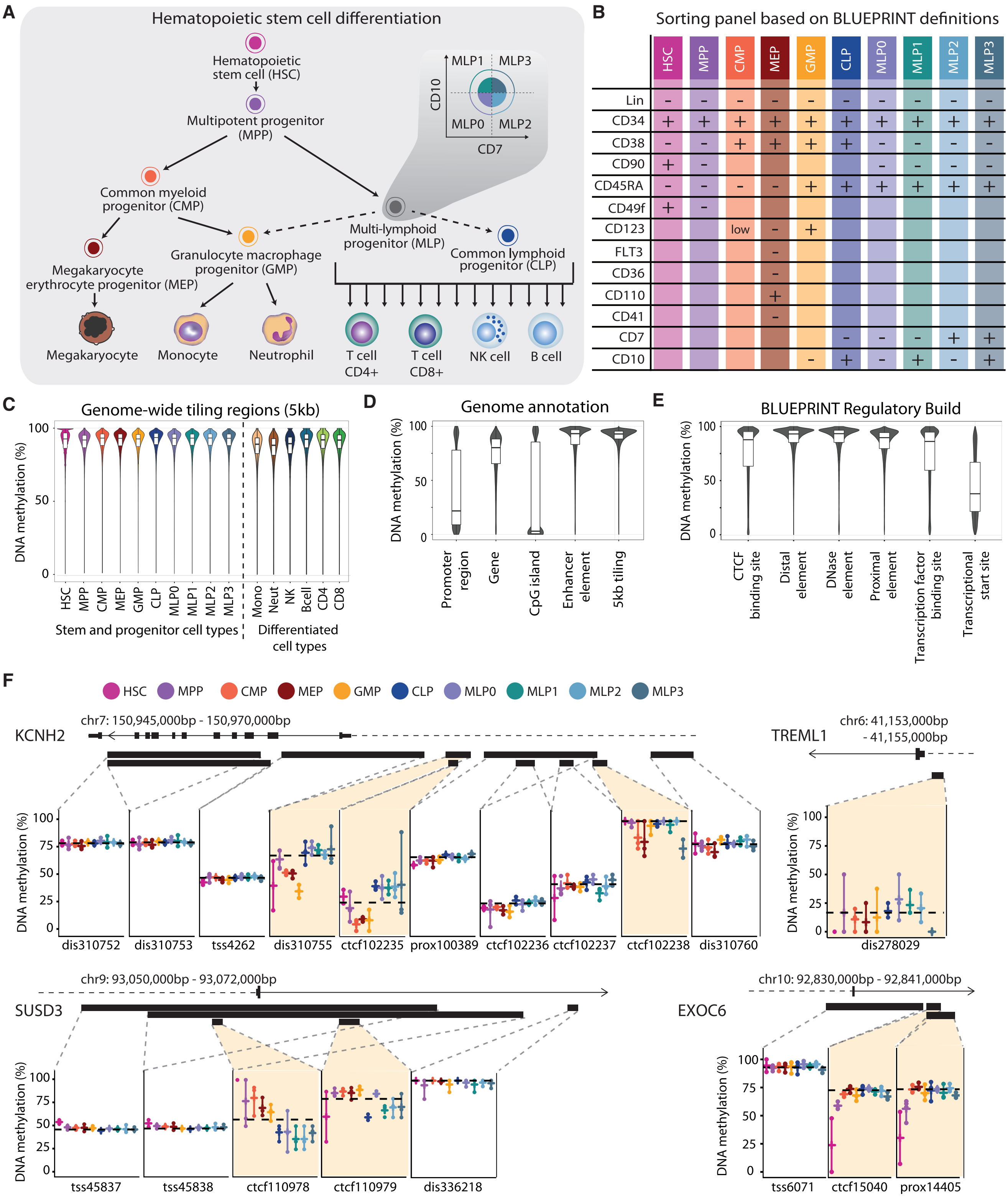

Figure 1. Charting the DNA Methylation Landscape of Human Hematopoietic Differentiation

(A) Conceptual outline of human hematopoietic differentiation, highlighting the 17 hematopoietic cell types whose genome-wide DNA methylation patterns were profiled in this study. Arrows denote established differentiation trajectories, dashed arrows indicate uncertainty about the in vivo differentiation potential of lymphoid progenitors, and the inset illustrates the sorting of four subsets of immature multi-lymphoid progenitors.

(B) Fluorescence-activated cell sorting panel used to purify 10 stem and progenitor cell types from peripheral blood.

(legend continued on next page)

the human blood lineage (Corces et al., 2016), and three recent studies used chromatin immunoprecipitation sequencing (ChIP-seq) to map histone modifications in the developing mouse embryo (Dahl et al., 2016; Liu et al., 2016; Zhang et al., 2016).

To establish a basis for epigenome-wide analysis and data-driven modeling of the human hematopoietic lineage, we applied our protocol for low-input and single-cell whole genome bisulfite sequencing (Farlik et al., 2015) to 17 hematopoietic cell types (Figure 1A). HSCs and multipotent progenitors (MPPs) were sorted from fetal liver, cord blood, bone marrow, and peripheral blood. Eight additional progenitor cell types and six differentiated cell types were sorted from peripheral blood, and megakaryocytes were sorted from bone marrow. For each stem and progenitor cell type, we sequenced an average of 32 low-input methylomes from three individuals, and we bioinformatically integrated them into meta-epigenomic profiles (Wijetunga et al., 2014). Additionally, we sequenced an average of 26 single-cell methylomes for seven cell types (HSC, MPP, common lymphoid progenitor [CLP], common myeloid progenitor [CMP], immature multi-lymphoid progenitor [MLP0], granulocyte macrophage progenitor [GMP], and megakaryocytes) to assess cell-to-cell heterogeneity.

Based on this dataset, which constitutes a community resource of the BLUEPRINT project (Adams et al., 2012) and the International Human Epigenome Consortium (IHEC; http://ihec-epigenomes.org), we compared DNA methylation between HSCs derived from different sources, and we studied changes in DNA methylation associated with commitment to the myeloid and lymphoid lineages. We also characterized novel subpopulations of immature multi-lymphoid progenitors and investigated the DNA methylation dynamics of megakaryocytes undergoing endomitotic replication. We linked the observed differences in DNA methylation to changes in gene expression, histone modifications, and chromatin accessibility, and we used machine learning to infer a model of human hematopoiesis directly from the DNA methylation data. These results highlight the power of DNA methylation analysis for in vivo dissection of cellular differentiation.

RESULTS

Comprehensive DNA Methylation Maps of Human Hematopoietic Stem and Progenitor Cell Types

We established fluorescence-activated cell sorting panels for 10 hematopoietic stem and progenitor cell types that are present in the peripheral blood of healthy individuals (Figures 1A and 1B; Supplemental Experimental Procedures). Each cell type was sorted from three donors to account for inter-individual heterogeneity. To enhance data quality for these rare cell types, we processed many small pools of cells in parallel and combined the results. Specifically, for each donor and cell type, we sorted and sequenced eight pools of 10 cells, two pools of 50 cells, and one pool of 1,000 cells (or a lower cell number where the target of 1,000 cells could not be reached).

DNA methylation libraries were generated by whole genome bisulfite sequencing (WGBS) using the μWGBS protocol (Farlik et al., 2015) and sequenced at low coverage to minimize the number of PCR duplicates. In total, 639 DNA methylation libraries passed quality control, and 3.1 terabases of sequencing data were produced (Table S1). DNA methylation profiles clustered predominantly by cell type (Figure S1A), indicating that neither technical biases arising from the different cell numbers nor inter-individual variation between donors had a strong influence on our investigation of cell-type-specific DNA methylation patterns. For further analysis, the DNA methylation profiles of all replicates of a given cell type were computationally combined into meta-epigenomic maps that provide consensus DNA methylation levels as well as an initial assessment of variability within cell types and among individuals.

The distribution of DNA methylation levels was similar across all stem and progenitor cell types, while we observed a shift toward lower levels in differentiated cells of the myeloid lineage (Figure 1C). Genome-wide DNA methylation patterns followed the well-established characteristics observed in mammalian genomes (Suzuki and Bird, 2008), including high levels of DNA methylation in most parts of the genome (as illustrated by 5-kb tiling regions) and locally reduced levels at gene promoters and CpG islands (Figure 1D).

To provide a robust and biologically meaningful basis for analyzing DNA methylation differences between cell types, we aggregated all DNA methylation data at the genomic region level based on the BLUEPRINT version of the Ensembl Regulatory Build (Zerbino et al., 2015). The BLUEPRINT Regulatory Build integrates epigenome data across many cell types into region sets that reflect the organizing principles of the human genome, thus facilitating the detection of meaningful DNA methylation differences (Bock, 2012). This catalog comprises six types of putative regulatory regions, which exhibit broadly varying DNA methylation levels in our dataset (Figure 1E).

The BLUEPRINT Regulatory Build also provides a framework for visualization (Figure 1F) and interactive analysis (http://blueprint-methylomes.computational-epigenetics.org) of DNA methylation at individual genomic loci. For example, two CTCF sites and a distal element inside the *KCNH2* gene (encoding a key factor for erythroid development) show decreased DNA methylation in the myeloid lineage, consistent with increased expression levels in CMP and GMP cells (Figure S1B). A putative enhancer of the myeloid-linked *TREML1* gene displays decreased DNA methylation in HSCs, MPPs, and myeloid

(C) Violin plots and boxplots showing the distribution of DNA methylation levels in 5-kb tiling regions for hematopoietic cell types sorted from peripheral blood.
(D) Distribution of DNA methylation levels across cell types for different sets of genomic regions. Gene and promoter annotations are based on GENCODE, CpG islands are from the UCSC Table Browser, enhancer elements are from Ensembl, and tiling regions were calculated with a custom script.
(E) Distribution of average DNA methylation levels across cell types for putative regulatory regions annotated by the Ensembl BLUEPRINT Regulatory Build.
(F) DNA methylation at putative regulatory regions for illustrative gene loci. Black bars denote the position of regions annotated by the BLUEPRINT Regulatory Build, and dashed horizontal black lines indicate sample medians for the respective regions. Colored vertical bars connect the highest and lowest DNA methylation levels that have been measured in any sample of the indicated cell type.
dis, distal element; prox, proximal element; TSS, transcriptional start site. See also Figure S1 and http://blueprint-methylomes.computational-epigenetics.org.

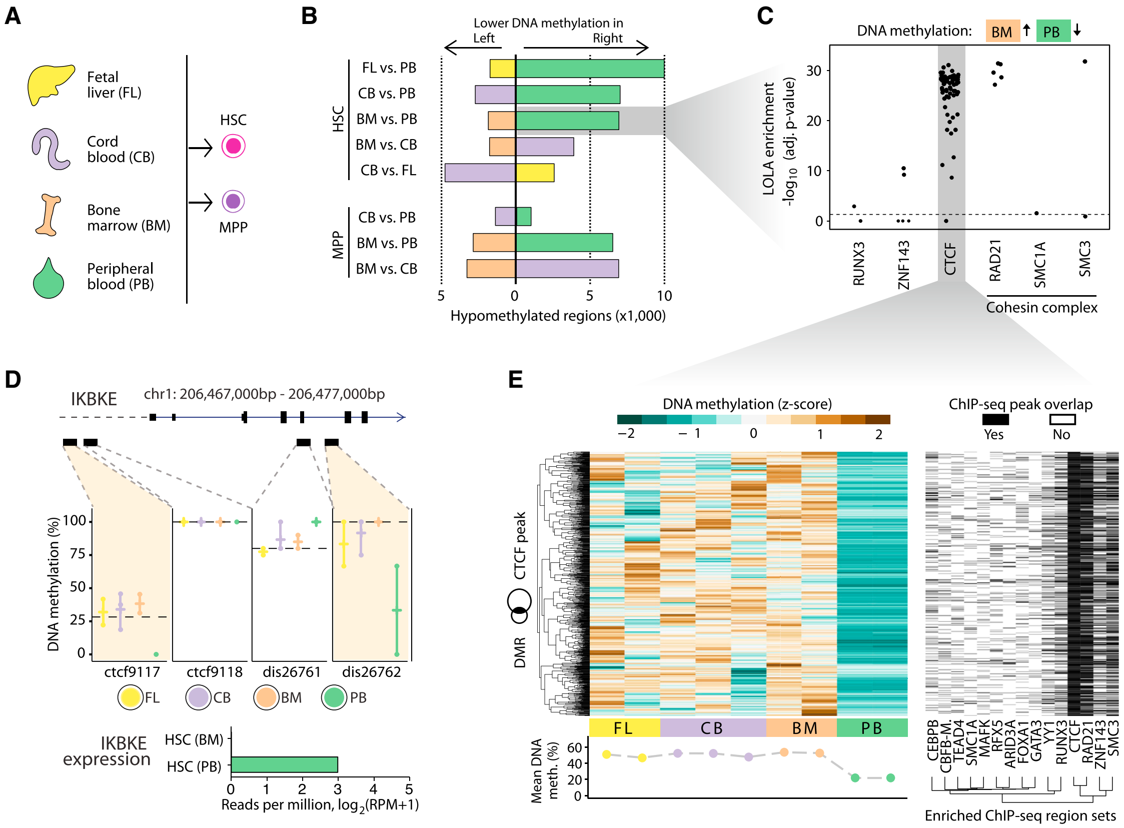

Figure 2. Comparison of DNA Methylation Maps for HSCs and MPPs Isolated from Four Different Sources

(A) HSCs and MPPs were sorted from the peripheral blood of three healthy donors (Figures 1A and 1B), and in addition from fetal liver (HSCs only), cord blood (HSCs and MPPs), and bone marrow (HSCs and MPPs).

(B) Bar plots showing the numbers of differentially methylated regions in pairwise comparisons between HSCs and MPPs from different sources, based on the BLUEPRINT Regulatory Build regions (FDR-adjusted $p \leq 0.05$, absolute difference ≥ 0.167 percentage points), calculated with RnBeads (Assenov et al., 2014).

(C) Region set enrichment analysis for genomic regions with lower DNA methylation in peripheral blood-derived HSCs compared with bone-marrow-derived HSCs. Enrichment was determined using LOLA (Sheffield and Bock, 2016). Each dot represents one ChIP-seq dataset, and the horizontal dashed line corresponds to a significance threshold of 0.05 on the adjusted p-value calculated by LOLA using Fisher's exact test.

(D) Source-specific DNA methylation at the *IKBKE* gene locus. Reduced DNA methylation levels in peripheral blood-derived HSC at two putative regulatory regions (BLUEPRINT Regulatory Build) is associated with detectable expression of the IKBKE gene specifically in this cell population (bar plot).

(E) Heatmap showing DNA methylation levels for regions that have lower DNA methylation in peripheral blood-derived HSCs than in bone-marrow-derived HSCs and also overlap with CTCF binding sites in the LOLA Core database (left). The second heatmap (right) shows the overlap of these regions with transcription factor binding sites that a LOLA analysis of this region set identified as enriched. Rows were arranged by hierarchical clustering with complete linkage based on the Euclidean distances between the DNA methylation profiles.

See also Figure S2 and http://blueprint-methylomes.computational-epigenetics.org.

progenitors, which correlates with increased RNA expression levels. CTCF sites in the lymphoid-linked *SUSD3* gene show lower DNA methylation in lymphoid progenitors, reflecting high expression in MLP0. Finally, a promoter-associated regulatory region in the *EXOC6* gene illustrates the frequently observed case of large DNA methylation differences that occur in the absence of detectable changes in gene expression.

DNA Methylation Distinguishes HSCs from Fetal Liver, Cord Blood, Bone Marrow, and Peripheral Blood

HSCs are rare in peripheral blood, whereas they exist in higher frequencies in fetal liver, cord blood, and bone marrow. HSCs obtained from these different sources have been shown to vary in their differentiation capacity (Notta et al., 2016), which prompted us to search for concomitant differences in their DNA methylation profiles. We obtained $CD34^+$ cells from fetal liver, cord blood, and bone marrow, and we sorted HSCs and MPPs in the same way as for peripheral blood (Figure 2A). DNA methylation analysis identified many more differences between peripheral blood and any of the other three sources (fetal liver, cord blood, and bone marrow) than between any two of the latter (Figure 2B; Table S2). Most of the genomic regions with source-dependent differences showed lower DNA methylation levels in HSCs and MPPs from peripheral blood, as compared with those obtained from the other sources.

We tested the regions that were specifically hypomethylated in peripheral blood HSCs for associations with transcription factor binding and regulatory elements using LOLA enrichment analysis (Sheffield and Bock, 2016). Significant overlap was observed with binding sites of CTCF, members of the cohesin

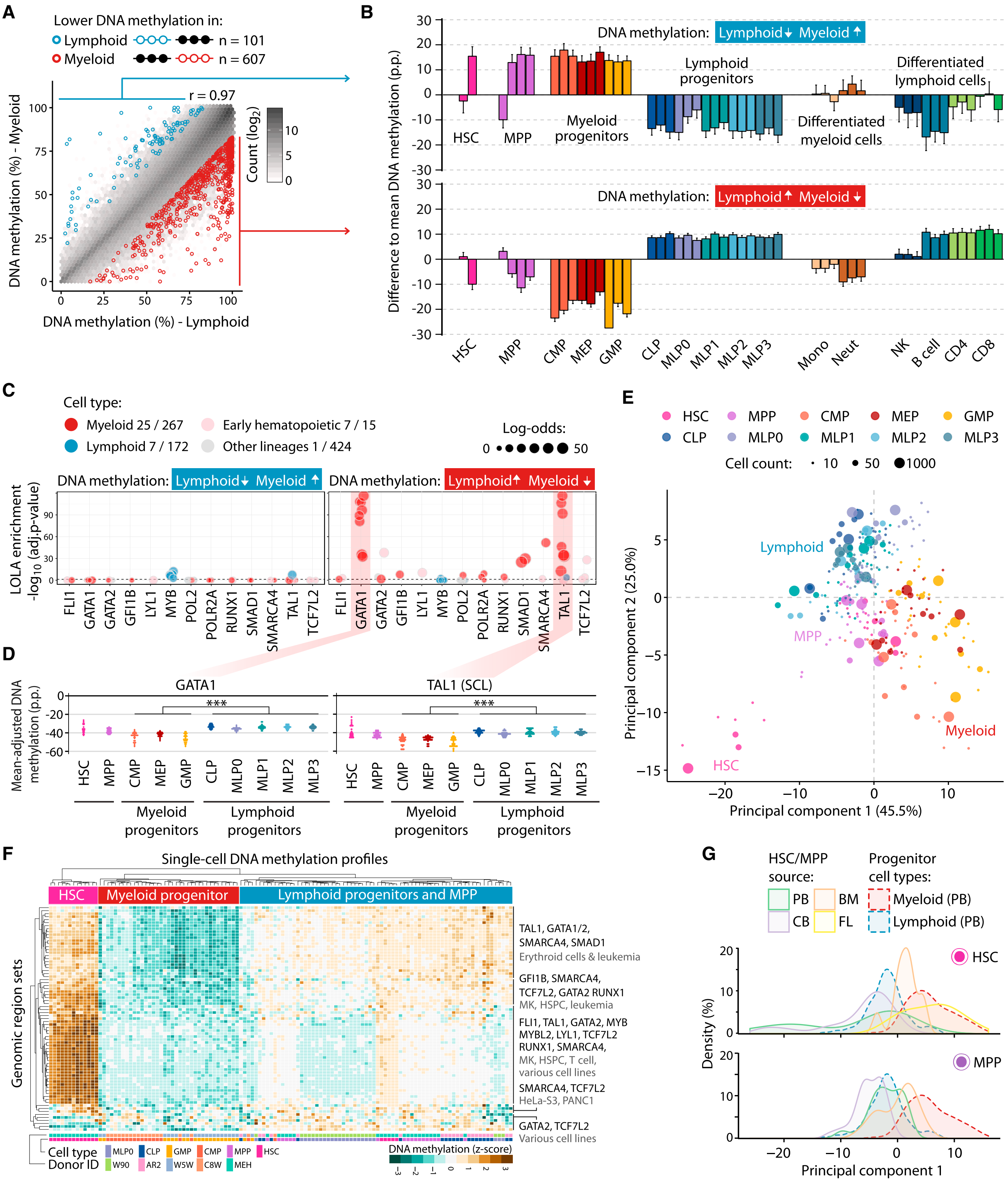

Figure 3. DNA Methylation Differences Associated with Myeloid-Lymphoid Lineage Commitment

(A) Scatterplot showing average DNA methylation levels for myeloid progenitors (CMP, MEP, GMP) and lymphoid progenitors (CLP, MLP0, MLP1, MLP2, MLP3) across BLUEPRINT Regulatory Build regions (Pearson's r = 0.97). Differentially methylated regions were identified with RnBeads (FDR-adjusted $p \leq 0.05$, absolute difference ≥ 0.167 percentage points).

(legend continued on next page)

complex (RAD21, SMC1A, SMC3), and the transcription factors RUNX3 and ZNF143 (Figure 2C; Table S3). We detected similar patterns for both HSCs and MPPs, whereas no such enrichment could be found, for example, for regions differentially methylated between MPPs in bone marrow and cord blood (Figure S2B). An illustrative example of peripheral blood hypomethylation of CTCF binding sites is given by the IKBKE gene (Figure 2D), which encodes a key kinase for NF-κB activation.

To identify additional transcription factors that may be associated with this CTCF-linked difference in DNA methylation, we performed LOLA analysis on all regions with lower DNA methylation in HSCs from peripheral blood than from bone marrow that also overlapped CTCF-bound regions (Figure 2E). This analysis confirmed the strong enrichment of cohesin complex proteins, while also detecting significant overlap for transcriptions factors relevant for hematopoietic development (FOXA1, GATA3, MAFK) and immune cell function (ARID3A, CEBPB, RFX5).

Myeloid-Lymphoid Lineage Choice Is Marked by DNA Methylation Depletion at Key Transcription Factor Binding Sites

After the initial transition from HSC to MPP, one major step of hematopoietic differentiation is the commitment to either the myeloid or the lymphoid lineage. DNA methylation levels at regulatory regions were on average lower in myeloid progenitors (CMP, megakaryocyte erythrocyte progenitor [MEP], GMP) than in lymphoid progenitors (MLP0, MLP1, MLP2, MLP3, CLP), and the same was true for differentiated cells of the two lineages (Figure S3A). Focusing again on the BLUEPRINT Regulatory Build (Figure 3A; Table S2), we also identified many more genomic regions with lower DNA methylation in myeloid cells (n = 607) than in lymphoid cells (n = 101). On average, these regions retained their differential DNA methylation in differentiated cells of the two lineages (Figure 3B).

Differentially methylated regions between myeloid and lymphoid progenitors were enriched for binding sites of 11 transcription factors and for RNA polymerase II binding in hematopoietic cells (Figure 3C; Table S3). The most striking overlap was observed between regions with lower DNA methylation in myeloid cells and binding sites of myeloerythroid transcription factors such as GATA1 and TAL1. In contrast, regions with lower DNA methylation levels in lymphoid progenitors did not show such strong enrichment patterns for any transcription factor binding sites annotated in the LOLA Core database. The average DNA methylation levels across all binding sites of the myeloid-specific transcription factors were reduced in myeloid progenitors when compared with lymphoid progenitors (Figures 3D and S3B). For about half of the transcription factors, the lower DNA methylation in myeloid (as opposed to lymphoid) progenitors was mirrored in higher expression levels in myeloid progenitors (Figure S3B).

The average DNA methylation depletion at the enriched transcription factor binding sites enabled consistent grouping of the individual replicates (10-, 50-, and 1,000-cell pools) according to their cellular lineage (Figure 3E), whereas the segregation by lineage was less clear when we performed the same analysis on all transcription factors in the LOLA Core database (Figure S3C). The first five principal components calculated from the mean-adjusted DNA methylation at the enriched transcription factor binding sites accounted for 82.3% of the observed variation (Figure S3D), whereas this value was much lower when focusing on all transcription factor binding sites (63.4%) or on all regions in the LOLA Core database (29.6%).

Averaging across pre-defined regulatory region sets is also a powerful method for analyzing single-cell data (Bock et al., 2016a; Farlik et al., 2015), and we applied this method to our set of 122 single-cell DNA methylation profiles comprising HSCs and MPPs, two myeloid progenitors (CMP and GMP), and two lymphoid progenitors (MLP0 and CLP). Plotting all single-cell profiles based on their mean-adjusted DNA methylation at enriched transcription factor binding sites (Figure 3F), we observed that region sets with low levels of DNA methylation in myeloid progenitors had much higher levels in HSCs. About half of these region sets were highly methylated in lymphoid progenitors, whereas the other half showed low levels of DNA methylation in some lymphoid progenitors (MLP0).

Finally, we investigated how the source-specific differences in DNA methylation among HSCs and MPPs (Figure 2) relate to differences between the myeloid and lymphoid lineage. To this end, we projected the DNA methylation data for HSCs and MPPs onto the first principal component identified in our analysis of mean-adjusted DNA methylation at transcription factor binding sites (Figure 3E), and we plotted the distribution along the first principal component, which was most informative for

(B) Average DNA methylation in each cell type relative to the mean over all samples aggregated over all regions with lower DNA methylation in lymphoid progenitors (top) and myeloid progenitors (bottom). Error bars correspond to the standard error.

(C) Region set enrichment analysis for regions with lower DNA methylation in lymphoid progenitors (left) or myeloid progenitors (right). Enrichment was determined using LOLA. Colored dots represent ChIP-seq experiments for transcription factors in the indicated lineage. Dot size denotes the log-odds ratio, and the numbers in the legend ("X/Y") refer to significantly enriched region sets (X) versus all analyzed region sets (Y). The horizontal dashed line represents the significance threshold (adjusted $p \leq 0.05$).

(D) Mean-adjusted DNA methylation relative to the average CpG methylation levels for each individual 10-, 50-, and 1,000-cell sample averaged across ChIP-seq peaks for GATA1 (left) and TAL1 (right). $^{***}p \leq 0.001$ (two-tailed Wilcoxon test).

(E) Two-dimensional projection of all 10-, 50-, and 1,000-cell samples from peripheral blood using principal component analysis based on the mean-adjusted DNA methylation across all transcription factor binding datasets identified by LOLA. The first two principal components are shown, and the numbers in parentheses indicate the percentage of variance explained.

(F) Heatmap displaying mean-adjusted DNA methylation for all single-cell DNA methylation profiles across the same region sets as in (E). Rows and columns are arranged by hierarchical clustering with Euclidean distance and complete linkage. The labels on the right summarize the transcription factors and cell types for the major branches of the row dendrogram.

(G) Distribution of HSC (top) and MPP (bottom) samples derived from fetal liver (FL), cord blood (CB), bone marrow (BM), and peripheral blood (PB) when projected onto the first principal component from (E).

p.p., percentage points. See also Figure S3 and http://blueprint-methylomes.computational-epigenetics.org.

the myeloid-lymphoid separation (Figure 3G). DNA methylation patterns in HSCs and MPPs from peripheral blood and cord blood were more similar to those of lymphoid progenitors, whereas cells from bone marrow and fetal liver showed higher similarity to myeloid progenitors.

Immature Multi-lymphoid Progenitors Show Characteristic DNA Methylation and Distinct Differentiation Propensities

Recent research identified a population of immature multi-lymphoid progenitors (MLPs) that may be ancestral to CLPs in the differentiation hierarchy (Doulatov et al., 2010, 2012; Goardon et al., 2011; Kohn et al., 2012). We sorted MLPs using the published set of surface markers (Doulatov et al., 2010) and further subdivided this cell population into four subtypes based on their CD10 and CD7 levels (Figures 4A and S4A).

To put the MLP subtypes into context with their differentiated progeny, we performed an unsupervised principal component analysis based on DNA methylation for all region sets contained in the LOLA Core database (Figure 4B). The first principal component segregated the MLP0 population ($CD10^-$, $CD7^-$) from the other progenitors and differentiated cell types. The second principal component discriminated between differentiated cell types of the myeloid and lymphoid lineage, placing the four MLP populations in an intermediate position.

We identified the region sets in the LOLA Core database that were most strongly associated with the first two principal components (Figure 4C). The first principal component comprised binding sites of broadly active transcription regulators and chromatin proteins (EP300, HDAC1, POL2, RBBP5, TAF1), whereas the second principal component included binding sites of transcription factors that are important for lymphoid and myeloid cell function (FOXA1, KAP1/TRIM28, MYC, STAT1, STAT3, TCF12).

We also assessed the differentiation capability of the lymphoid progenitors using in vitro colony formation assays (Figures 4D and S4B). CLPs from peripheral blood gave rise not only to lymphoid-restricted colonies, but also to a small number of myeloid and mixed myeloid and lymphoid colonies. This is in contrast with a previous analysis of cord-blood-derived cells (Doulatov et al., 2010) and highlights that the differentiation potential of progenitor populations in human is dependent on the cell source and stage of ontogeny (Notta et al., 2016). All MLP populations displayed higher proportions of mixed myeloid and lymphoid colonies than observed for the CLPs. The differentiation potential was similar among the four MLP subtypes, although MLP0 gave rise to the smallest number of myeloid-only colonies (Figure 4D) and had the highest potential for B cells and granulocytes (Figure 4E).

Endomitotic Replication of Megakaryocytes Is Accompanied by Progressive Changes in DNA Methylation Patterns

In the myeloid lineage, megakaryocyte maturation involves endomitotic replication and an exponential increase in cell ploidy (Figure 5A). Megakaryocytes are thought to be derived from MEPs, although evidence for mouse and human suggests an alternative origin directly from HSCs (Haas et al., 2015; Notta et al., 2016; Sanjuan-Pla et al., 2013). We collected megakaryocytes from the bone marrow of three donors, sorted them according to their ploidy (2N, 4N, 8N, 16N, 32N), and performed DNA methylome sequencing on 61 single cells and ten 5-cell pools (Table S1). The results were highly consistent between the single-cell and 5-cell samples (Figures S5A and S5B), arguing against technical biases caused by different DNA amounts influencing our analysis.

Comparing DNA methylation for all LOLA Core region sets between diploid (2N) and polyploid (32N) megakaryocytes, we observed strong correlation (Pearson's $r = 0.99$) and highly similar distributions of DNA methylation values (Figures 5B, S5A, and S5B), indicating that megakaryocyte maturation does not involve any large genome-wide changes in DNA methylation as previously observed for mouse erythroblast maturation (Shearstone et al., 2011). Nevertheless, a small number of region sets were differentially methylated, and these regions underwent consistent and progressive changes across the different ploidy stages of megakaryocyte maturation (Figure 5C).

Progressively increasing DNA methylation levels were observed for DNase I hypersensitive sites specific to hematopoietic cells and for binding sites of NFE2, which is a regulator of megakaryocyte maturation and platelet production (Lecine et al., 1998). Conversely, decreasing DNA methylation occurred in DNase I hypersensitive sites from a broader set of cell types and at the binding sites of hematopoietic transcription factors with an established role in megakaryocyte-erythroblast differentiation, including GATA1, SMAD1, and TAL1 (Tijssen et al., 2011).

The region sets that showed progressively decreasing DNA methylation levels in maturing megakaryocytes were on average more highly methylated in other progenitor cell types than in megakaryocytes (Figure S5C). In contrast, region sets with progressively increasing DNA methylation levels during megakaryocyte maturation moved toward the average levels in other progenitors rather than away from it.

DNA Methylation Differences Are Linked to Cell-type-Specific Transcription Levels and Chromatin Signatures

DNA methylation at gene promoters can be associated with transcriptional repression, although the genome-wide correlation between DNA methylation and gene expression is low (Jones, 2012; Suzuki and Bird, 2008). To investigate this association in our dataset, we generated RNA-seq data for 100-cell pools of stem and progenitor cell types sorted from peripheral blood (Table S1) (http://blueprint-methylomes.computational-epigenetics.org), and we identified 656 genes that were differentially expressed between myeloid and lymphoid progenitors (false discovery rate [FDR]-adjusted $p \leq 0.05$, $|\log_2 FC| \geq 1$). Gene Ontology analysis revealed an enrichment for genes associated with lymphocyte function in lymphoid progenitors and for genes associated with hemostasis in myeloid progenitors (Figures 6A and 6B).

When we linked the observed differences in gene expression to DNA methylation differences at associated promoters, we found only a small number of genes with strong and concordant changes (Figure 6C), which is consistent with previous observations for mouse hematopoiesis (Bock et al., 2012). Among the genes whose promoters were less methylated and more highly expressed in myeloid progenitors were myeloid regulators such as TAL1, MYB, MARCKS, and ICAM4. Conversely, several

CellPress

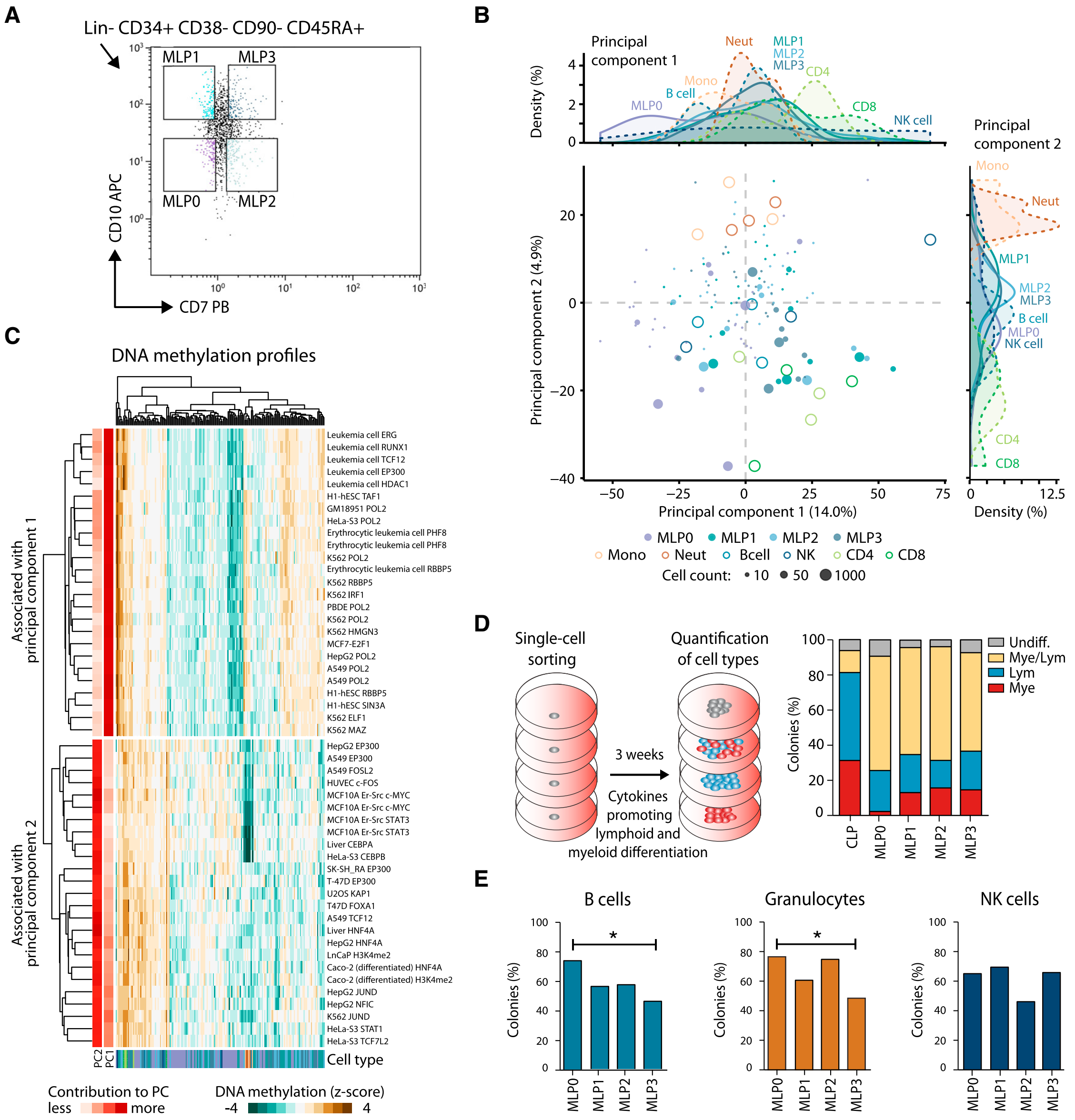

Figure 4. Characterization of MLP Populations by DNA Methylation and In Vitro Differentiation Assays

(A) Sorting panel for purifying four MLP populations from peripheral blood.

(B) Two-dimensional projection of all 10-, 50-, and 1,000-cell MLP samples using principal component analysis based on the mean-adjusted DNA methylation relative to the average CpG methylation levels across all region sets in the LOLA Core database. The first two principal components are shown, the numbers in parentheses indicate the percentage of variance explained, and the density plots (top and right) summarize the distribution of cell types along the two principal components.

(C) Heatmap displaying the mean-adjusted DNA methylation for all MLP samples across the 2 × 25 genomic region sets that contributed most strongly to the first principal component (PC1, top) and the second principal component (PC2, bottom). Rows and columns are arranged by hierarchical clustering with Euclidean distance and complete linkage. The row labels indicate the cell type and ChIP-seq target of the corresponding LOLA region sets.

(D) Differentiation potential of CLPs and four MLP populations measured by in vitro culture of single cells on MS-5 stroma with cytokines promoting lymphoid and myeloid differentiation. The percentage of colonies that show lymphoid ($CD19^+$ or $CD56^+$) as well as myeloid ($CD14^+$ or $CD15^+$) markers was determined by flow cytometry.

(E) Differentiation potential of the MLP populations measured as the percentage of colonies containing B cells ($CD19^+$), granulocytes ($CD15^+$), or NK cells ($CD56^+$) in flow cytometry. *$p \leq 0.05$ (Fisher's exact test).

See also Figure S4 and http://blueprint-methylomes.computational-epigenetics.org.

genes that play a role in lymphocyte function—including ITGAL, DUSP1, and MX1—were less methylated and more highly expressed in lymphoid progenitors (Figure 6C).

We also investigated the link between DNA methylation and histone modifications. Using ChIP-seq profiles for differentiated blood cells types, which have been generated as part of the

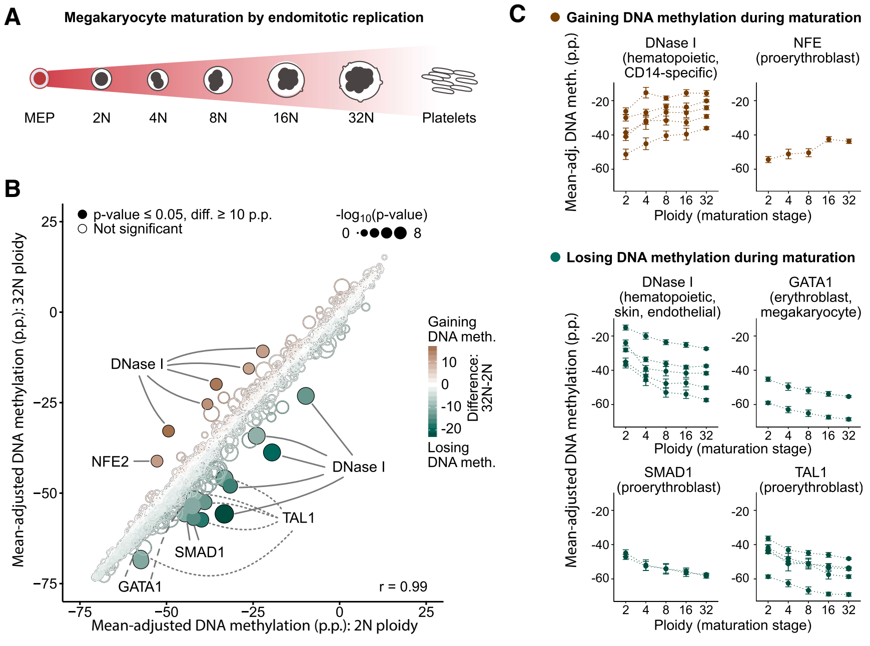

Figure 5. DNA Methylation Analysis of Megakaryocyte Maturation

(A) Conceptual outline of megakaryocyte development from MEPs via a maturation phase involving endomitotic genome replication and a concomitant increase in ploidy.

(B) Scatterplot comparing mean-adjusted DNA methylation (relative to the average CpG methylation level in each sample) for all region sets in the LOLA Core database between megakaryocyte at the 2N and at the 32N stage of ploidy. Region sets that were significantly less methylated in 32N ($n = 14$, bottom right) or in 2N megakaryocyte ($n = 6$, top left) are highlighted with filled circles ($p \leq 0.05$, Wilcoxon test, absolute difference $\geq$ 10 p.p.). Point colors indicate the magnitude of difference, and the size is proportional to statistical significance [$-\log_{10}(p)$].

(C) Mean-adjusted DNA methylation in region sets that gain (top) or lose DNA methylation (bottom) as identified in (B), plotted across ploidy stages of megakaryocyte maturation. Error bars correspond to the standard error.

p.p., percentage points. See also Figure S5 and http://blueprint-methylomes.computational-epigenetics.org.

BLUEPRINT project (Adams et al., 2012), we calculated consensus maps for three histone modifications (H3K4me1, H3K27ac, and H3K27me3) in myeloid and lymphoid cells. Regions with lower DNA methylation levels in myeloid progenitors showed higher H3K4me1 levels in differentiated myeloid cells, and the opposite was true for regions with lower DNA methylation in lymphoid progenitors (Figure 6D). For H3K27ac, we observed consistently higher levels in lymphoid cells than in myeloid cells, whereas the observed differences for H3K27me3 were less pronounced than for the other marks.

Finally, we compared our DNA methylation data with a recently published chromatin accessibility dataset (Corces et al., 2016). This dataset includes ATAC-seq profiles for several hematopoietic stem and progenitor cell types, from which we derived cell-type-specific regions of open chromatin. Genomic regions with HSC-specific open chromatin had low DNA methylation levels across all cell types (Figures 6E–6G, S6A, and S6B), regions with open chromatin in differentiated cells showed reduced DNA methylation levels only in the corresponding cell type while being highly methylated in progenitors, and regions with accessible chromatin in myeloid or lymphoid progenitors were hypomethylated only in differentiated cells of the respective lineage.

Computational Modeling Identifies Predictive Epigenetic Signatures that Support Data-Driven Lineage Reconstruction

Having identified characteristic DNA methylation dynamics in several branches of the human hematopoietic lineage, we employed machine learning methods in order to predict cell types from DNA methylation patterns, to quantify epigenetic similarity, and to infer cellular differentiation landscapes. We based this analysis on classifiers that were trained to predict cell type from genome-wide DNA methylation profiles in putative regulatory regions (Figure 7A).

Specifically, we used elastic net-regularized general linear models (Friedman et al., 2010) for predicting the cell type of each individual stem and progenitor sample in our dataset. These classifiers were trained on the DNA methylation levels of all BLUEPRINT Regulatory Build regions in each 10-, 50-, and 1,000-cell sample, and the model performance was evaluated using 10-fold cross-validation (Figures 7B and S7A).

We observed high prediction accuracies for all cell types, with receiver operating characteristic (ROC) area under curve (AUC) values for individual cell types ranging from 0.85 to 1.00 (Figure S7A). Highest accuracies were obtained for myeloid progenitors (CMP, GMP, and MEP) and for the MLP0 population. Lymphoid progenitors (CLP, MLP1, MLP2, and MLP3) were more difficult to distinguish, consistent with their similar DNA methylation profiles (Figure S1A) and similar functional properties (Figure 4E). Lowest AUC values were observed for the HSC and MLP2 cell populations, which were frequently confused with MPPs and CLPs, respectively (Figure 7B).

The regularized classifiers weigh all genomic regions by their discriminatory power, thus establishing a measure of their importance for cell-type prediction. Based on this measure, we identified a set of 1,234 signature regions whose DNA methylation levels collectively distinguished hematopoietic cell types with high accuracy and robustness (Figure 7C; Table S4). Individual DNA methylation differences were small for most of these regions, highlighting that many weak but complementary differences can support accurate cell-type prediction.

LOLA enrichment analysis for the signature regions identified significant overlap with the binding sites of key hematopoietic transcription factors such as FLI1, GATA1/2, MYB, RUNX1, and TAL1 (Figure 7D). Unsupervised analysis based on the signature regions identified strong separation between myeloid and lymphoid progenitors, but no clear clustering within each group (Figure 7E). Moreover, differentiated cell types of the

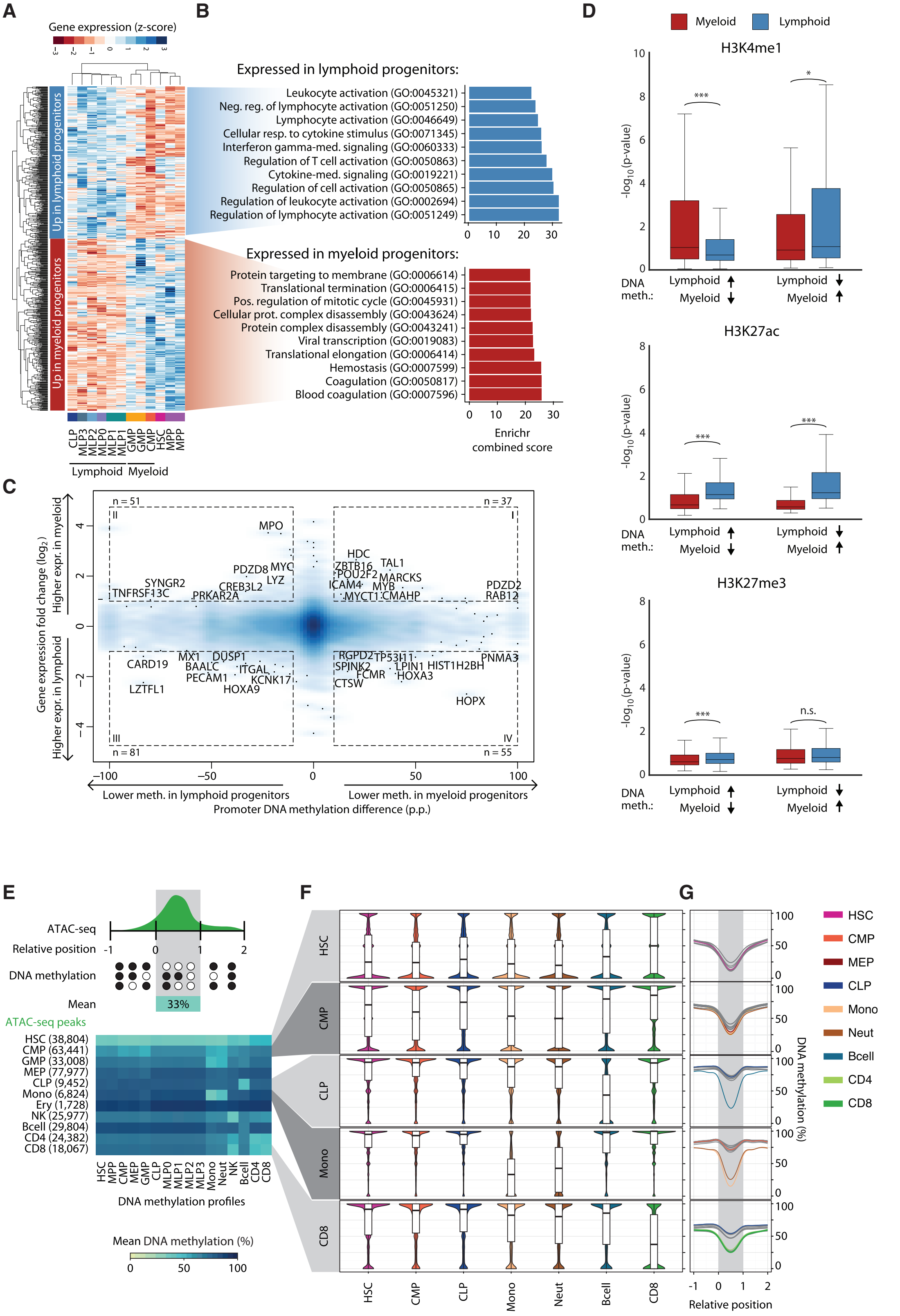

(legend on next page)

myeloid and lymphoid lineage formed separate clusters in the vicinity of their corresponding progenitors.

To quantify the similarity between cell types, we trained 10 additional classifiers, each excluding one of the stem and progenitor cell types (“leave-one-out-classifiers”), and we calculated the class probabilities for the samples that were withheld from the analysis (see Experimental Procedures). These class probabilities (Figure S7B; Table S5) define a data-driven network model of the human hematopoietic lineage, which emerges from the characteristic DNA methylation patterns of each cell type and their relationship with each other (Figure 7F).

DISCUSSION

We established genome-wide maps of the DNA methylation dynamics in human hematopoietic differentiation, which comprise 17 cell types, four different sources of HSCs, and a total of 639 DNA methylation profiles. This resource, accessible via public repositories and a dedicated website (http://blueprint-methylomes.computational-epigenetics.org), provides insights into the role of epigenetic regulation in HSCs and their differentiating progeny, and it constitutes a reference for biomedical research focusing on diseases of the blood.

A key outcome of our study is the high accuracy with which DNA methylation profiles predict cell type throughout the human hematopoietic lineage. This is not merely due to the correlation between DNA methylation and gene expression (which was low in our dataset), but rather suggests that DNA methylation itself reflects a cell’s differentiation trajectory at the epigenetic level. We showed that prediction based on DNA methylation in regulatory regions can place sorted cell populations into a developmental context. DNA methylation analysis thus complements studies of human hematopoietic differentiation that were based on gene expression profiling (Chen et al., 2014; Notta et al., 2016; Novershtern et al., 2011) and chromatin accessibility mapping (Corces et al., 2016).

To illustrate the value of our dataset for biological hypothesis generation and for guiding mechanistic studies on specific aspects of hematopoietic differentiation, we focused on four areas of the human hematopoietic lineage.

First, we compared HSCs from four different sources. Peripheral blood is readily accessible and therefore highly relevant for clinical diagnostics. To establish a broadly useful reference, we thus based most of our dataset on stem and progenitor cell populations purified from the peripheral blood of healthy donors. Nevertheless, the microenvironment of peripheral blood differs markedly from that of bone marrow, cord blood, and fetal liver, which are commonly used sources of HSCs in basic research. HSCs from peripheral blood showed lower DNA methylation levels at the binding sites of CTCF and cohesin complex proteins than HSCs from other sources, which may reflect changes in chromatin 3D architecture that influence gene expression. These differences stress the importance of taking cell source and microenvironment into account when studying human hematopoietic stem and progenitor cells.

Second, we investigated the DNA methylation dynamics of myeloid-lymphoid lineage choice, observing an asymmetric pattern: regulatory regions that showed reduced DNA methylation levels in myeloid progenitors were enriched for binding sites of transcription factors associated with hematopoietic differentiation, myeloid lineage fate, and leukemia as well as lymphoma, whereas there was no strong enrichment among regions that had reduced DNA methylation levels in lymphoid progenitors. This observation is consistent with DNA methylation data for mouse hematopoiesis (Bock et al., 2012), and together with the finding that lymphoid differentiation is compromised in transgenic mice with impaired maintenance DNA methylation (Bröske et al., 2009), it supports the view that DNA methylation may epigenetically shield lymphoid progenitors from the default program of myeloid differentiation.

Third, we combined DNA methylation mapping and in vitro differentiation assays to characterize four populations of immature multi-lymphoid progenitors that appear to constitute epigenetically and functionally distinguishable cell types. MLP0 ($CD7^{-}$ $CD10^{-}$) showed the most distinctive DNA methylation signature and highest levels of multi-lineage differentiation potential from individual cells. The observed patterns of multi-lineage differentiation among MLPs and CLPs may reflect an underappreciated level of epigenetic plasticity in human hematopoietic differentiation (Notta et al., 2016; Paul et al., 2015).

Figure 6. Integrative Analysis of Gene Expression, Histone Modifications, and Chromatin Accessibility

(A) Heatmap showing row-normalized expression levels for 656 differentially expressed genes (FDR-adjusted $p \leq 0.05$, $|\log_2 FC| \geq 1$) between myeloid progenitors (CMP, GMP) and lymphoid progenitors (CLP, MLP0, MLP1, MLP2, MLP3) determined using DEseq2 (Love et al., 2014). Rows and columns were arranged by hierarchical clustering with Euclidean distance and complete linkage.

(B) Top 10 most highly enriched Gene Ontology terms associated with genes overexpressed in lymphoid (top) and myeloid (bottom) progenitors based on the Enrichr software (Kuleshov et al., 2016).

(C) Density scatterplot contrasting myeloid-lymphoid differences in DNA methylation at gene promoters with expression differences of the corresponding genes. Selected genes with strong differences in DNA methylation (absolute difference $\geq$ 10 p.p.) and gene expression ($|\log_2 FC| \geq 1$) are highlighted.

(D) Boxplots showing histone modification levels for open chromatin-associated H3K4me1, active enhancer-linked H3K27ac, and polycomb-associated H3K27me3 in regions that were differentially methylated between myeloid and lymphoid progenitors (Figure 3A). Histone modification levels were calculated from multiple ChIP-seq datasets for myeloid cells (neutrophils, monocytes, and macrophages, in red) and lymphoid cells (NK cells, B cells, and $CD4^{+}/CD8^{+}$ T cells, in blue). Brackets identify two-tailed Mann-Whitney U tests. $^{*}p \leq 0.05$, $^{***}p \leq 0.001$.

(E) Heatmap showing DNA methylation levels (columns) in regions with cell-type-specific chromatin accessibility based on published ATAC-seq data for hematopoietic cell types (rows). Numbers in parentheses denote the number of chromatin accessible regions specific to each cell type.

(F) Distribution of DNA methylation levels across regions with cell-type-specific chromatin accessibility.

(G) Composite plots showing DNA methylation averages across regions with cell-type-specific chromatin accessibility. CpGs in the neighborhood of these regions were annotated with coordinates relative to their start and end (x axis). CpGs with a relative coordinate of 0 and 1 are located at the start and end of a region, respectively, and the coordinates −1 and 2 correspond to one region length upstream and downstream of the region. The curves show cubic spline smoothing of DNA methylation levels per cell type across accessible regions.

p.p., percentage points; n.s., not significant. See also Figure S6 and http://blueprint-methylomes.computational-epigenetics.org.

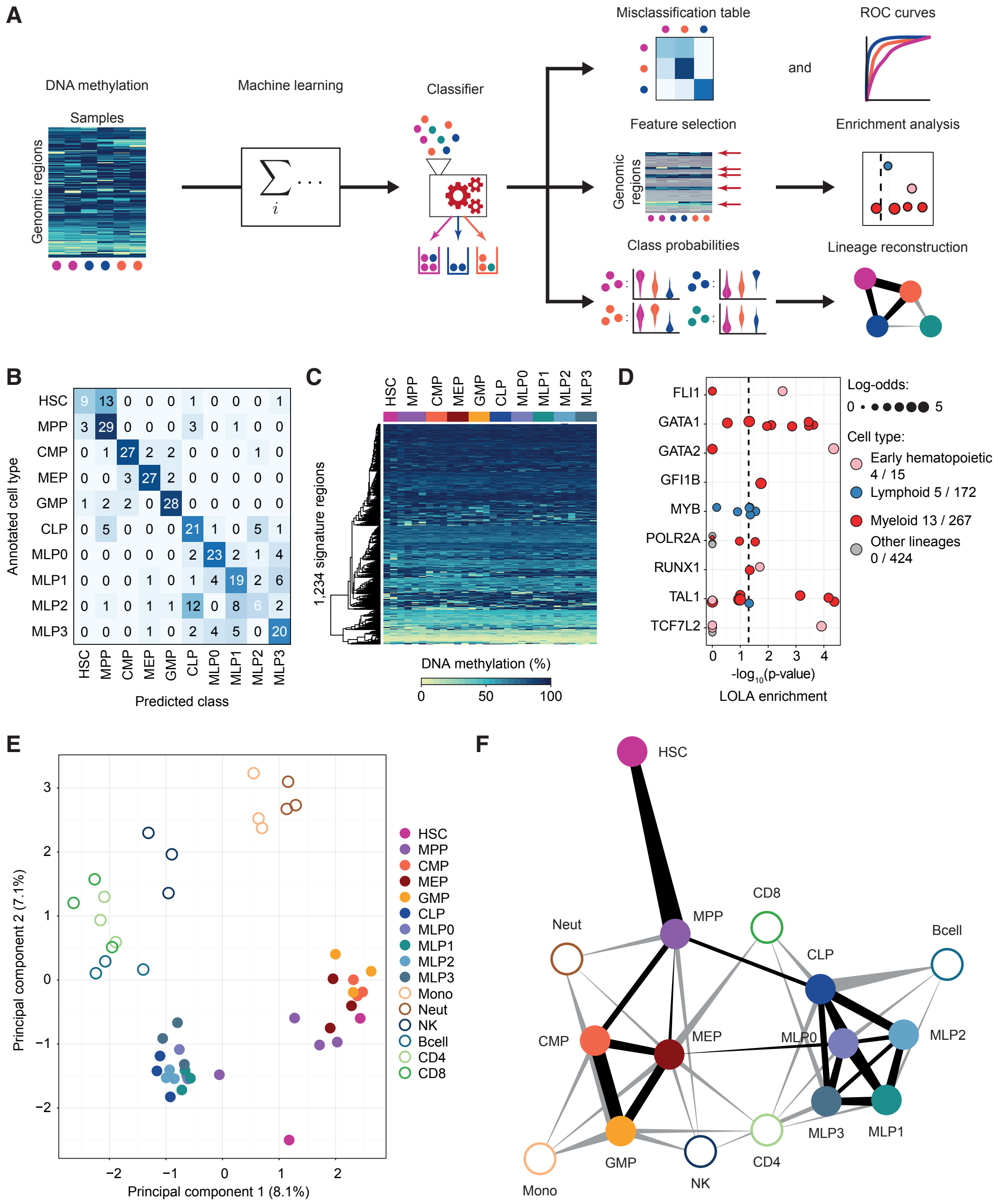

Figure 7. Data-Driven Reconstruction of the Human Hematopoietic Lineage using Machine Learning

(A) Conceptual outline of the machine learning approach used to predict cell type, to identify signature regions, and to infer cellular differentiation landscapes.

(B) Confusion matrix showing the frequency of misclassification based on 10-fold cross-validation of cell-type classifiers trained and evaluated on 319 stem and progenitor samples (all 10-, 50-, and 1,000-cell pools) from peripheral blood.

(C) Heatmap showing average DNA methylation levels of merged replicates (one column for each cell type in each donor) for the 1,234 signature regions extracted from a classifier trained on all peripheral blood-derived stem and progenitor samples. Regions (rows) were arranged using hierarchical clustering with Euclidean distance and complete linkage.

(D) Region set enrichment analysis for the signature regions using LOLA. Colored dots represent ChIP-seq experiments for transcription factors in the indicated lineage. Dot size denotes the log-odds ratio, and the numbers in the legend ("X/Y") refer to significantly enriched region sets (X) versus all analyzed region sets (Y). The vertical dashed line represents the significance threshold (adjusted $p \leq 0.05$).

(E) Two-dimensional projection of merged replicates (one point for each cell type in each donor) using principal component analysis based on average DNA methylation levels in the signature regions. The first two principal components are shown, and the numbers in parentheses indicate the percentage of variance explained.

(F) Hematopoietic lineage reconstruction using the prediction propensities of DNA methylation-based classifiers as a measure of similarity between cell types. Nodes in the graph represent cell types, and edges are weighted by class probabilities of cross-prediction. An automated edge-weighted graph layout algorithm was used to define the positions of the nodes.

See also Figure S7 and http://blueprint-methylomes.computational-epigenetics.org.

Fourth, we analyzed the DNA methylation dynamics over the course of megakaryocyte maturation, which involves multiple rounds of endomitotic replication and consequent increases in ploidy. Whereas the cellular morphology of maturing megakaryocytes changes dramatically, DNA methylation levels at regulatory regions showed only mild, but consistent and progressive, changes. Certain genomic regions (counter-intuitively including NFE2 binding sites) started off with low levels in 2N megakaryocytes but gained DNA methylation up to a level comparable with HSCs, whereas the majority of region sets started with myeloid-like DNA methylation levels that were lost during maturation.

In summary, we have established a comprehensive catalog of DNA methylation in human hematopoietic differentiation, which provides a resource and framework for studying the different cell types of the blood, as well as their associated diseases. Given the medical relevance (Laird, 2003) and technical feasibility (Bock et al., 2016b) of using DNA methylation as a clinical biomarker, it is expected that detailed DNA methylation analysis of immunodeficiencies, cardiovascular diseases, and blood cell malignancies will help advance precision medicine.

EXPERIMENTAL PROCEDURES

Sample Preparation Summary

Peripheral blood cells were isolated from apheresis filters of healthy platelet donors belonging to the National Institute for Health Research (NIHR) Cambridge BioResource after informed consent and with ethical approval (REC 12/EE/0040). Cells were stained with antibodies and sorted on either BD Influx or BD FACSAria III fluorescence-activated cell sorting instruments. Library preparation followed the μWGBS/scWGBS protocol as described previously (Farlik et al., 2015). A detailed description of the sample collection, purification, library preparation, and sequencing is provided in the Supplemental Experimental Procedures.

Data Analysis Summary

Bisulfite sequencing reads were aligned with Bismark v0.12.2 (Krueger and Andrews, 2011) and processed with RnBeads v1.5 (Assenov et al., 2014) to aggregate DNA methylation values on regulatory regions annotated by the August 2015 release of the BLUEPRINT Ensembl Regulatory Build (Zerbino et al., 2015). Elastic net-regularized general linear models implemented in the R package glmnet (Krishnapuram et al., 2005) were used for cell-type prediction, and the cell-type similarity graph (Figure 7F) was derived from average class probabilities assigned by leave-one-class-out classifiers trained separately for each cell type. A detailed description of the sequencing data processing, differential DNA methylation analysis, genomic region enrichment analysis, single-cell DNA methylation analysis, and cell-type prediction is provided in the Supplemental Experimental Procedures.

Data Availability and Accession Numbers

The presented dataset can be accessed through five alternative and complementary sources:

1. A supplemental website with additional diagrams and tables, which also contains direct links to the other data sources, is available at http://blueprint-methylomes.computational-epigenetics.org.
2. The genome browser track hub, which is linked at http://blueprint-methylomes.computational-epigenetics.org, provides the processed DNA methylation data for interactive visualization and processing with online tools such as Galaxy.
3. Preprocessed data (DNA methylation calls and gene expression levels) can be downloaded without any restrictions from GEO: GSE87197.
4. The raw sequencing data from which the DNA methylation calls and gene expression levels have been derived are available from the European Genome-phenome Archive (EGA): EGAS00001002070 (controlled access to protect patient privacy).
5. The dataset is included in the epigenome registry of IHEC (http://www.ebi.ac.uk/vg/epirr, accession numbers IHECRE00002734 to IHECRE00002810), the DeepBlue Epigenomic Data Server (http://deepblue.mpi-inf.mpg.de), and the IHEC Data Portal (http://epigenomesportal.ca/ihec).

SUPPLEMENTAL INFORMATION

Supplemental Information includes Supplemental Experimental Procedures, seven figures, and five tables and can be found with this article online at http://dx.doi.org/10.1016/j.stem.2016.10.019.

AUTHOR CONTRIBUTIONS

Conceptualization, M. Farlik, F.H., F.M., H.G.S., M. Frontini, and C.B.; Methodology, M. Farlik, F.H., F.M., M. Frontini, and C.B.; Formal analysis, F.H., F.M., P.E., and J.K.; Investigation, M. Farlik, F.A.C., S.F., A.S., V.C., A.M., and R.U.; Writing – Original Draft, M. Farlik, F.H., F.M., P.E., M. Frontini, and C.B.; Writing – Review & Editing, F.A.C., J.K., S.F., A.S., V.C., A.M., R.U., H.G.S., W.H.O, E.L., and T.L.; Supervision, E.L., T.L., M. Frontini, and C.B.; Funding acquisition, H.G.S., W.H.O., E.L., T.L., M. Frontini, and C.B.

ACKNOWLEDGMENTS

This work was performed in the context of, and on behalf of, the BLUEPRINT project. We gratefully acknowledge the participation of NIHR Cambridge BioResource volunteers, and we thank the Cambridge BioResource staff for help with volunteer recruitment. We also thank Emily F. Calderbank and the Cambridge Biomedical Research Centre's Cell Phenotyping Hub for contributions to cell purification, Thomas Krausgruber for help with data visualization, Nathan C. Sheffield and André F. Rendeiro for providing bioinformatic methods and software, Sebastian Ullrich and Roderic Guigó for help with RNA data processing, Paul Flicek and his team for providing the BLUEPRINT Regulatory Build, the members of the Cambridge BioResource Scientific Advisory Board and Management Committee for their support, the Biomedical Sequencing Facility at CeMM for assistance with next generation sequencing, and many members of the BLUEPRINT and IHEC consortia for helpful advice and generous data sharing. This work was funded by the BLUEPRINT project (European Union's Seventh Framework Programme grant 282510), the NIHR Cambridge Biomedical Research Centre, and the Austrian Academy of Sciences. F.A.C. is supported by a Medical Research Council Clinical Training Fellowship (grant MR/K024043/1). F.H. is supported by a postdoctoral fellowship of the German Research Council (DFG; grant HA 7723/1-1). J.K. is supported by a DOC Fellowship of the Austrian Academy of Sciences. W.H.O. is supported by the NIHR, BHF (grants PG-0310-1002 and RG/09/12/28096), and NHS Blood and Transplant. E.L. is supported by a Wellcome Trust Sir Henry Dale Fellowship (grant 107630/Z/15/Z) and core support grant from the Wellcome Trust and MRC to the Wellcome Trust-Medical Research Council Cambridge Stem Cell Institute. M. Frontini is supported by the BHF Cambridge Centre of Excellence (grant RE/13/6/30180). C.B. is supported by a New Frontiers Group award of the Austrian Academy of Sciences and by a European Research Council (ERC) Starting Grant (European Union's Horizon 2020 research and innovation program; grant 679146).

Received: February 19, 2016
Revised: October 4, 2016
Accepted: October 24, 2016
Published: November 17, 2016

REFERENCES

Adams, D., Altucci, L., Antonarakis, S.E., Ballesteros, J., Beck, S., Bird, A., Bock, C., Boehm, B., Campo, E., Caricasole, A., et al. (2012). BLUEPRINT to decode the epigenetic signature written in blood. Nat. Biotechnol. *30*, 224–226.

Assenov, Y., Müller, F., Lutsik, P., Walter, J., Lengauer, T., and Bock, C. (2014). Comprehensive analysis of DNA methylation data with RnBeads. Nat. Methods *11*, 1138–1140.

Bock, C. (2012). Analysing and interpreting DNA methylation data. Nat. Rev. Genet. *13*, 705–719.

Bock, C., Kiskinis, E., Verstappen, G., Gu, H., Boulting, G., Smith, Z.D., Ziller, M., Croft, G.F., Amoroso, M.W., Oakley, D.H., et al. (2011). Reference Maps of human ES and iPS cell variation enable high-throughput characterization of pluripotent cell lines. Cell *144*, 439–452.

Bock, C., Beerman, I., Lien, W.H., Smith, Z.D., Gu, H., Boyle, P., Gnirke, A., Fuchs, E., Rossi, D.J., and Meissner, A. (2012). DNA methylation dynamics during in vivo differentiation of blood and skin stem cells. Mol. Cell *47*, 633–647.

Bock, C., Farlik, M., and Sheffield, N.C. (2016a). Multi-omics of single cells: strategies and applications. Trends Biotechnol. *34*, 605–608.

Bock, C., Halbritter, F., Carmona, F.J., Tierling, S., Datlinger, P., Assenov, Y., Berdasco, M., Bergmann, A.K., Booher, K., Busato, F., et al.; BLUEPRINT Consortium (2016b). Quantitative comparison of DNA methylation assays for biomarker development and clinical applications. Nat. Biotechnol. *34*, 726–737.

Bröske, A.-M., Vockentanz, L., Kharazi, S., Huska, M.R., Mancini, E., Scheller, M., Kuhl, C., Enns, A., Prinz, M., Jaenisch, R., et al. (2009). DNA methylation protects hematopoietic stem cell multipotency from myeloerythroid restriction. Nat. Genet. *41*, 1207–1215.

Cabezas-Wallscheid, N., Klimmeck, D., Hansson, J., Lipka, D.B., Reyes, A., Wang, Q., Weichenhan, D., Lier, A., von Paleske, L., Renders, S., et al. (2014). Identification of regulatory networks in HSCs and their immediate progeny via integrated proteome, transcriptome, and DNA methylome analysis. Cell Stem Cell *15*, 507–522.

Chen, L., Kostadima, M., Martens, J.H., Canu, G., Garcia, S.P., Turro, E., Downes, K., Macaulay, I.C., Bielczyk-Maczynska, E., Coe, S., et al.; BRIDGE Consortium (2014). Transcriptional diversity during lineage commitment of human blood progenitors. Science *345*, 1251033.

Corces, M.R., Buenrostro, J.D., Wu, B., Greenside, P.G., Chan, S.M., Koenig, J.L., Snyder, M.P., Pritchard, J.K., Kundaje, A., Greenleaf, W.J., et al. (2016). Lineage-specific and single-cell chromatin accessibility charts human hematopoiesis and leukemia evolution. Nat. Genet. *48*, 1193–1203.

Dahl, J.A., Jung, I., Aanes, H., Greggains, G.D., Manaf, A., Lerdrup, M., Li, G., Kuan, S., Li, B., Lee, A.Y., et al. (2016). Broad histone H3K4me3 domains in mouse oocytes modulate maternal-to-zygotic transition. Nature *537*, 548–552.

Doulatov, S., Notta, F., Eppert, K., Nguyen, L.T., Ohashi, P.S., and Dick, J.E. (2010). Revised map of the human progenitor hierarchy shows the origin of macrophages and dendritic cells in early lymphoid development. Nat. Immunol. *11*, 585–593.

Doulatov, S., Notta, F., Laurenti, E., and Dick, J.E. (2012). Hematopoiesis: a human perspective. Cell Stem Cell *10*, 120–136.

Farlik, M., Sheffield, N.C., Nuzzo, A., Datlinger, P., Schönegger, A., Klughammer, J., and Bock, C. (2015). Single-cell DNA methylome sequencing and bioinformatic inference of epigenomic cell-state dynamics. Cell Rep. *10*, 1386–1397.

Fernandez, A.F., Assenov, Y., Martin-Subero, J.I., Balint, B., Siebert, R., Taniguchi, H., Yamamoto, H., Hidalgo, M., Tan, A.C., Galm, O., et al. (2012). A DNA methylation fingerprint of 1628 human samples. Genome Res. *22*, 407–419.

Friedman, J., Hastie, T., and Tibshirani, R. (2010). Regularization paths for generalized linear models via coordinate descent. J. Stat. Softw. *33*, 1–22.

Goardon, N., Marchi, E., Atzberger, A., Quek, L., Schuh, A., Soneji, S., Woll, P., Mead, A., Alford, K.A., Rout, R., et al. (2011). Coexistence of LMPP-like and GMP-like leukemia stem cells in acute myeloid leukemia. Cancer Cell *19*, 138–152.

Haas, S., Hansson, J., Klimmeck, D., Loeffler, D., Velten, L., Uckelmann, H., Wurzer, S., Prendergast, Á.M., Schnell, A., Hexel, K., et al. (2015). Inflammation-induced emergency megakaryopoiesis driven by hematopoietic stem cell-like megakaryocyte progenitors. Cell Stem Cell *17*, 422–434.

Habibi, E., Brinkman, A.B., Arand, J., Kroeze, L.I., Kerstens, H.H.D., Matarese, F., Lepikhov, K., Gut, M., Brun-Heath, I., Hubner, N.C., et al. (2013). Whole-genome bisulfite sequencing of two distinct interconvertible DNA methylomes of mouse embryonic stem cells. Cell Stem Cell *13*, 360–369.

Ji, H., Ehrlich, L.I.R., Seita, J., Murakami, P., Doi, A., Lindau, P., Lee, H., Aryee, M.J., Irizarry, R.A., Kim, K., et al. (2010). Comprehensive methylome map of lineage commitment from haematopoietic progenitors. Nature *467*, 338–342.

Jones, P.A. (2012). Functions of DNA methylation: islands, start sites, gene bodies and beyond. Nat. Rev. Genet. *13*, 484–492.

Kim, K., Zhao, R., Doi, A., Ng, K., Unternaehrer, J., Cahan, P., Huo, H., Loh, Y.H., Aryee, M.J., Lensch, M.W., et al. (2011). Donor cell type can influence the epigenome and differentiation potential of human induced pluripotent stem cells. Nat. Biotechnol. *29*, 1117–1119.

Kohn, L.A., Hao, Q.-L., Sasidharan, R., Parekh, C., Ge, S., Zhu, Y., Mikkola, H.K.A., and Crooks, G.M. (2012). Lymphoid priming in human bone marrow begins before expression of CD10 with upregulation of L-selectin. Nat. Immunol. *13*, 963–971.

Krishnapuram, B., Carin, L., Figueiredo, M.A.T., and Hartemink, A.J. (2005). Sparse multinomial logistic regression: fast algorithms and generalization bounds. IEEE Trans. Pattern Anal. Mach. Intell. *27*, 957–968.

Krueger, F., and Andrews, S.R. (2011). Bismark: a flexible aligner and methylation caller for Bisulfite-Seq applications. Bioinformatics *27*, 1571–1572.

Kuleshov, M.V., Jones, M.R., Rouillard, A.D., Fernandez, N.F., Duan, Q., Wang, Z., Koplev, S., Jenkins, S.L., Jagodnik, K.M., Lachmann, A., et al. (2016). Enrichr: a comprehensive gene set enrichment analysis web server 2016 update. Nucleic Acids Res. *44* (W1), W90–W97.

Kundaje, A., Meuleman, W., Ernst, J., Bilenky, M., Yen, A., Heravi-Moussavi, A., Kheradpour, P., Zhang, Z., Wang, J., Ziller, M.J., et al.; Roadmap Epigenomics Consortium (2015). Integrative analysis of 111 reference human epigenomes. Nature *518*, 317–330.

Laird, P.W. (2003). The power and the promise of DNA methylation markers. Nat. Rev. Cancer *3*, 253–266.

Lecine, P., Villeval, J.L., Vyas, P., Swencki, B., Xu, Y., and Shivdasani, R.A. (1998). Mice lacking transcription factor NF-E2 provide in vivo validation of the proplatelet model of thrombocytopoiesis and show a platelet production defect that is intrinsic to megakaryocytes. Blood *92*, 1608–1616.

Lister, R., Mukamel, E.A., Nery, J.R., Urich, M., Puddifoot, C.A., Johnson, N.D., Lucero, J., Huang, Y., Dwork, A.J., Schultz, M.D., et al. (2013). Global epigenomic reconfiguration during mammalian brain development. Science *341*, 1237905.

Liu, X., Wang, C., Liu, W., Li, J., Li, C., Kou, X., Chen, J., Zhao, Y., Gao, H., Wang, H., et al. (2016). Distinct features of H3K4me3 and H3K27me3 chromatin domains in pre-implantation embryos. Nature *537*, 558–562.

Love, M.I., Huber, W., and Anders, S. (2014). Moderated estimation of fold change and dispersion for RNA-seq data with DESeq2. Genome Biol. *15*, 550.

McCracken, M.N., Gschweng, E.H., Nair-Gill, E., McLaughlin, J., Cooper, A.R., Riedinger, M., Cheng, D., Nosala, C., Kohn, D.B., and Witte, O.N. (2013). Long-term in vivo monitoring of mouse and human hematopoietic stem cell engraftment with a human positron emission tomography reporter gene. Proc. Natl. Acad. Sci. USA *110*, 1857–1862.

Moran, S., Martínez-Cardús, A., Sayols, S., Musulén, E., Balañá, C., Estival-Gonzalez, A., Moutinho, C., Heyn, H., Diaz-Lagares, A., de Moura, M.C., et al. (2016). Epigenetic profiling to classify cancer of unknown primary: a multicentre, retrospective analysis. Lancet Oncol. *17*, 1386–1395.

Notta, F., Zandi, S., Takayama, N., Dobson, S., Gan, O.I., Wilson, G., Kaufmann, K.B., McLeod, J., Laurenti, E., Dunant, C.F., et al. (2016). Distinct routes of lineage development reshape the human blood hierarchy across ontogeny. Science *351*, aab2116.

Novershtern, N., Subramanian, A., Lawton, L.N., Mak, R.H., Haining, W.N., McConkey, M.E., Habib, N., Yosef, N., Chang, C.Y., Shay, T., et al. (2011). Densely interconnected transcriptional circuits control cell states in human hematopoiesis. Cell *144*, 296–309.

CellPress

Park, C.Y., Majeti, R., and Weissman, I.L. (2008). In vivo evaluation of human hematopoiesis through xenotransplantation of purified hematopoietic stem cells from umbilical cord blood. Nat. Protoc. *3*, 1932–1940.

Paul, F., Arkin, Y., Giladi, A., Jaitin, D.A., Kenigsberg, E., Keren-Shaul, H., Winter, D., Lara-Astiaso, D., Gury, M., Weiner, A., et al. (2015). Transcriptional heterogeneity and lineage commitment in myeloid progenitors. Cell *163*, 1663–1677.

Polo, J.M., Liu, S., Figueroa, M.E., Kulalert, W., Eminli, S., Tan, K.Y., Apostolou, E., Stadtfeld, M., Li, Y., Shioda, T., et al. (2010). Cell type of origin influences the molecular and functional properties of mouse induced pluripotent stem cells. Nat. Biotechnol. *28*, 848–855.

Sanjuan-Pla, A., Macaulay, I.C., Jensen, C.T., Woll, P.S., Luis, T.C., Mead, A., Moore, S., Carella, C., Matsuoka, S., Bouriez Jones, T., et al. (2013). Platelet-biased stem cells reside at the apex of the haematopoietic stem-cell hierarchy. Nature *502*, 232–236.

Shearstone, J.R., Pop, R., Bock, C., Boyle, P., Meissner, A., and Socolovsky, M. (2011). Global DNA demethylation during mouse erythropoiesis in vivo. Science *334*, 799–802.

Sheffield, N.C., and Bock, C. (2016). LOLA: enrichment analysis for genomic region sets and regulatory elements in R and Bioconductor. Bioinformatics *32*, 587–589.

Smallwood, S.A., Tomizawa, S., Krueger, F., Ruf, N., Carli, N., Segonds-Pichon, A., Sato, S., Hata, K., Andrews, S.R., and Kelsey, G. (2011). Dynamic CpG island methylation landscape in oocytes and preimplantation embryos. Nat. Genet. *43*, 811–814.

Smith, Z.D., Chan, M.M., Mikkelsen, T.S., Gu, H., Gnirke, A., Regev, A., and Meissner, A. (2012). A unique regulatory phase of DNA methylation in the early mammalian embryo. Nature *484*, 339–344.

Spangrude, G.J. (1991). Hematopoietic stem-cell differentiation. Curr. Opin. Immunol. *3*, 171–178.

Suzuki, M.M., and Bird, A. (2008). DNA methylation landscapes: provocative insights from epigenomics. Nat. Rev. Genet. *9*, 465–476.

Tanner, A., Taylor, S.E., Decottignies, W., and Berges, B.K. (2014). Humanized mice as a model to study human hematopoietic stem cell transplantation. Stem Cells Dev. *23*, 76–82.

Theocharides, A.P.A., Rongvaux, A., Fritsch, K., Flavell, R.A., and Manz, M.G. (2016). Humanized hemato-lymphoid system mice. Haematologica *101*, 5–19.

Tijssen, M.R., Cvejic, A., Joshi, A., Hannah, R.L., Ferreira, R., Forrai, A., Bellissimo, D.C., Oram, S.H., Smethurst, P.A., Wilson, N.K., et al. (2011). Genome-wide analysis of simultaneous GATA1/2, RUNX1, FLI1, and SCL binding in megakaryocytes identifies hematopoietic regulators. Dev. Cell *20*, 597–609.

Till, J.E., and McCulloch, E.A. (1980). Hemopoietic stem cell differentiation. Biochim. Biophys. Acta *605*, 431–459.

Vedi, A., Santoro, A., Dunant, C.F., Dick, J.E., and Laurenti, E. (2016). Molecular landscapes of human hematopoietic stem cells in health and leukemia. Ann. N Y Acad. Sci. *1370*, 5–14.

Wijetunga, N.A., Delahaye, F., Zhao, Y.M., Golden, A., Mar, J.C., Einstein, F.H., and Greally, J.M. (2014). The meta-epigenomic structure of purified human stem cell populations is defined at cis-regulatory sequences. Nat. Commun. *5*, 5195.

Woolthuis, C.M., and Park, C.Y. (2016). Hematopoietic stem/progenitor cell commitment to the megakaryocyte lineage. Blood *127*, 1242–1248.

Zerbino, D.R., Wilder, S.P., Johnson, N., Juettemann, T., and Flicek, P.R. (2015). The Ensembl Regulatory Build. Genome Biol. *16*, 56.

Zhang, B., Zheng, H., Huang, B., Li, W., Xiang, Y., Peng, X., Ming, J., Wu, X., Zhang, Y., Xu, Q., et al. (2016). Allelic reprogramming of the histone modification H3K4me3 in early mammalian development. Nature *537*, 553–557.

Ziller, M.J., Gu, H., Müller, F., Donaghey, J., Tsai, L.T.-Y., Kohlbacher, O., De Jager, P.L., Rosen, E.D., Bennett, D.A., Bernstein, B.E., et al. (2013). Charting a dynamic DNA methylation landscape of the human genome. Nature *500*, 477–481.

Cell Stem Cell, Volume *19*

Supplemental Information

DNA Methylation Dynamics of Human Hematopoietic Stem Cell Differentiation

Matthias Farlik, Florian Halbritter, Fabian Müller, Fizzah A. Choudry, Peter Ebert, Johanna Klughammer, Samantha Farrow, Antonella Santoro, Valerio Ciaurro, Anthony Mathur, Rakesh Uppal, Hendrik G. Stunnenberg, Willem H. Ouwehand, Elisa Laurenti, Thomas Lengauer, Mattia Frontini, and Christoph Bock

Supplemental Figures

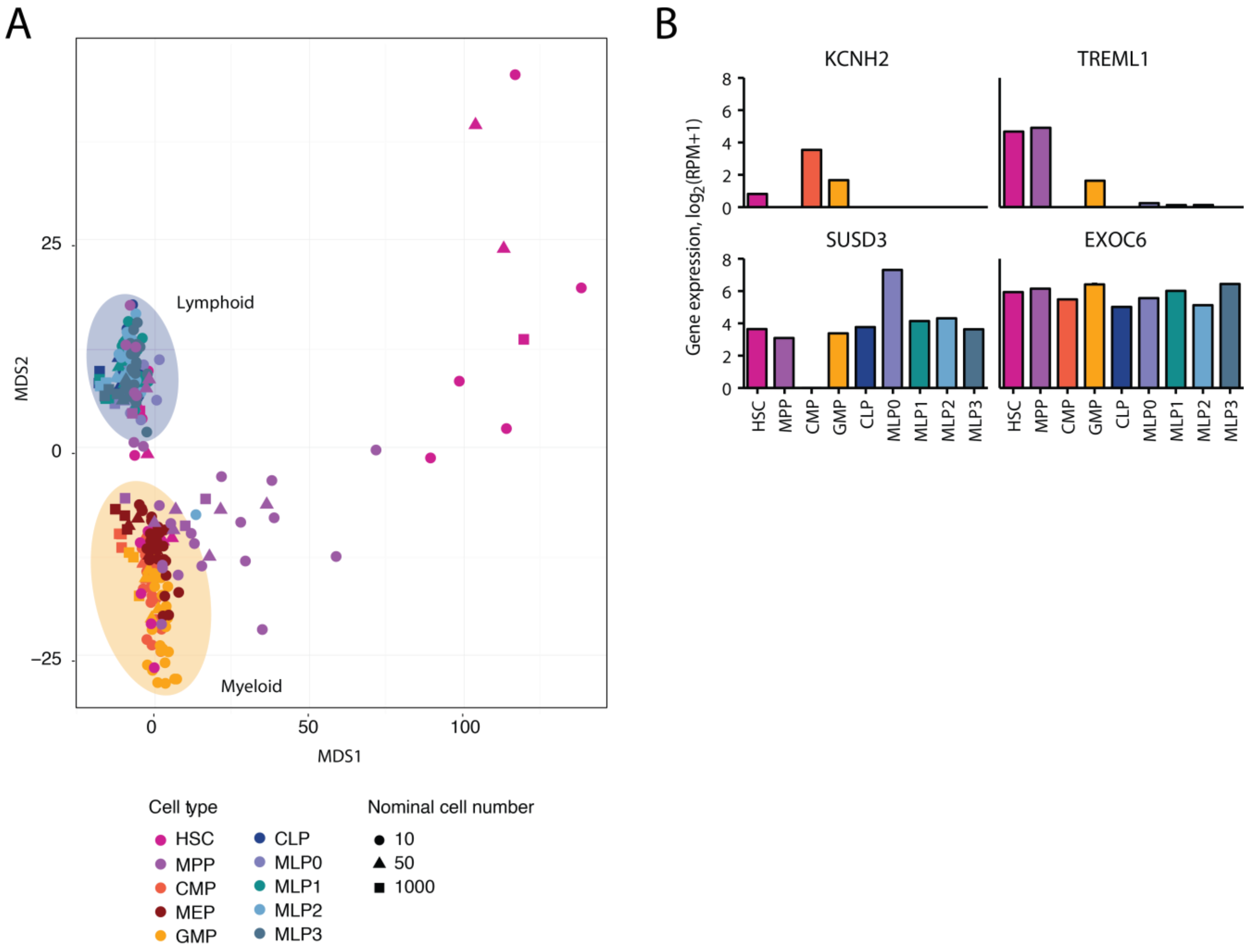

Figure S1. DNA methylation and gene expression profiles of blood stem and progenitor cells

A) Unsupervised multidimensional scaling (MDS) analysis of DNA methylation profiles for 10-cell, 50-cell, and 1,000-cell samples of hematopoietic stem and progenitor cell types sorted from peripheral blood. DNA methylation levels were aggregated at region level based on the BLUEPRINT Regulatory Build. The analysis results are dominated by two compact clusters comprising lymphoid and myeloid cells, while HSC and MPP profiles are separated and more dispersed. The number of cells in each pool did not have a strong effect on the grouping.

B) Gene expression levels of KCNH2, TREML1, SUSD3, and EXOC6 in the indicated stem and progenitor cell types measured by RNA-seq.

Related to Figure 1.

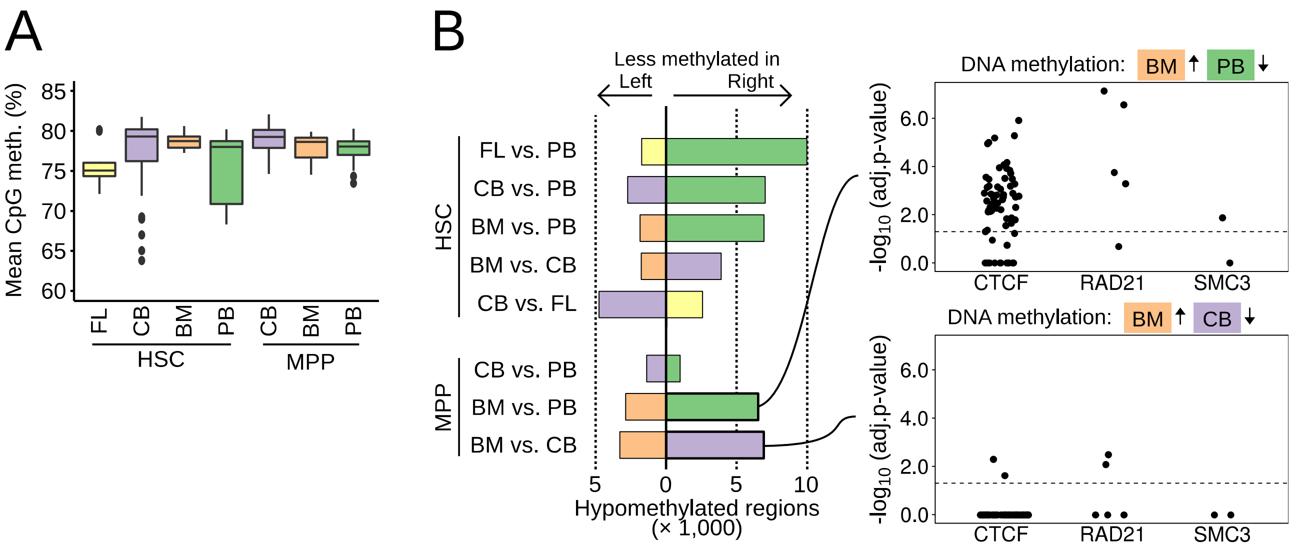

Figure S2. DNA methylation differences for HSCs and MPPs isolated from different sources

A) Distribution of average CpG methylation levels for HSCs (left) and MPPs (right) isolated from different sources. FL: fetal liver, CB: cord blood, BM: bone marrow, PB: peripheral blood.

B) Enrichment of CTCF, RAD21, and SMC3 binding sites for regions with lower DNA methylation in peripheral blood-derived MPPs than in bone-marrow-derived MPPs (left), or with lower DNA methylation in cord blood-derived MPPs than in bone-marrow-derived MPPs (right). Enrichment was determined using LOLA (Sheffield and Bock, 2016). Each dot represents one ChIP-seq dataset, and the dashed line corresponds to a significance threshold of 0.05 on the adjusted p-value calculated by LOLA using Fisher's exact test. Enrichment p-values were high for comparisons that involved peripheral blood-derived HSCs (**Figure 2C**) and peripheral blood-derived MPPs (top right), while they were lower in other comparisons of similar size (bottom right).

Related to Figure 2.

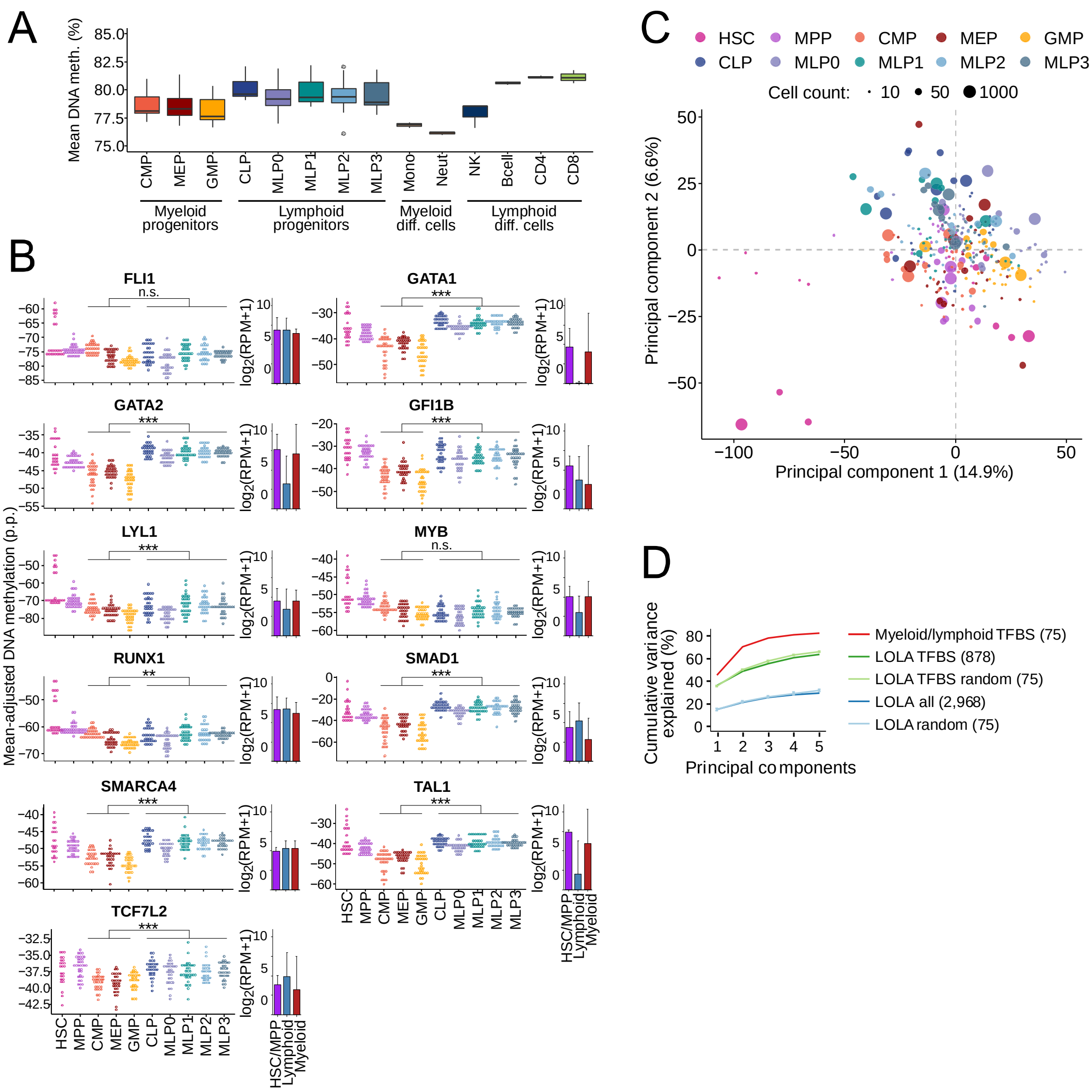

Figure S3. DNA methylation differences between myeloid and lymphoid progenitors

A) Distribution of average DNA methylation levels across BLUEPRINT Regulatory Build regions in progenitors and differentiated cell types of the myeloid and lymphoid lineages.

B) Mean-adjusted DNA methylation relative to the average CpG methylation levels for each individual 10-cell, 50-cell, and 1,000-cell sample averaged across ChIP-seq peaks for all enriched transcription factors shown in **Figure 3C**. The bar plots on the right of each diagram show the average gene expression levels of the corresponding transcription factors in HSCs/MPPs, in lymphoid progenitors (CLP, MLP0, MLP1, MLP2, MLP3), and in myeloid progenitors (CMP, MEP, GMP). Error bars correspond to the standard error. Brackets indicate two-tailed Wilcoxon tests with FDR-adjusted p-values. ***: $p \leq 0.001$, **: $p \leq 0.01$, n.s.: $p \geq 0.05$, p.p.: percentage points.

C) Two-dimensional projection of all 10-cell, 50-cell, and 1,000-cell samples from peripheral blood using principal component analysis based on the mean-adjusted DNA methylation across all 2,968 ChIP-seq region sets in the LOLA Core database. The first two principal components are shown, and the numbers in parentheses indicate the percentage of variance explained.

D) Cumulative percentage of variance explained by the first five principal components calculated from the mean-adjusted DNA methylation across all regions in the LOLA Core database (blue line), across 75 randomly selected datasets from this database averaged over 100 random samplings (light blue line), across all transcription factor binding sites (TFBS) from ENCODE and CODEX (green line), across 75 randomly selected datasets from these databases averaged over 100 random samplings (light green line), or across 75 transcription factor binding sites relevant to myeloid/lymphoid differentiation (red line) as in **Figure 3C**.

Related to Figure 3.

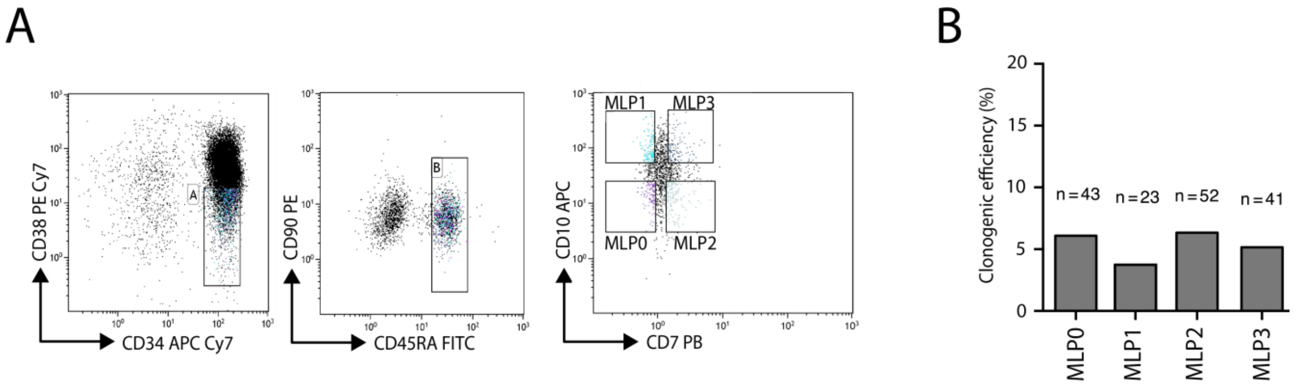

Figure S4. Sorting and *in vitro* differentiation of immature multi-lymphoid progenitors

A) Immature multi-lymphoid progenitor cells (MLP0, MLP1, MLP2, MLP3) were sorted from the CD34+, CD45RA+ fraction of peripheral blood based on the expression of CD10 and CD7.

B) Bar plots summarizing the clonogenic efficiency determined by *in vitro* colony formation assays for the four MLP populations. The total number of tested cells of each type is indicated.

Related to Figure 4.

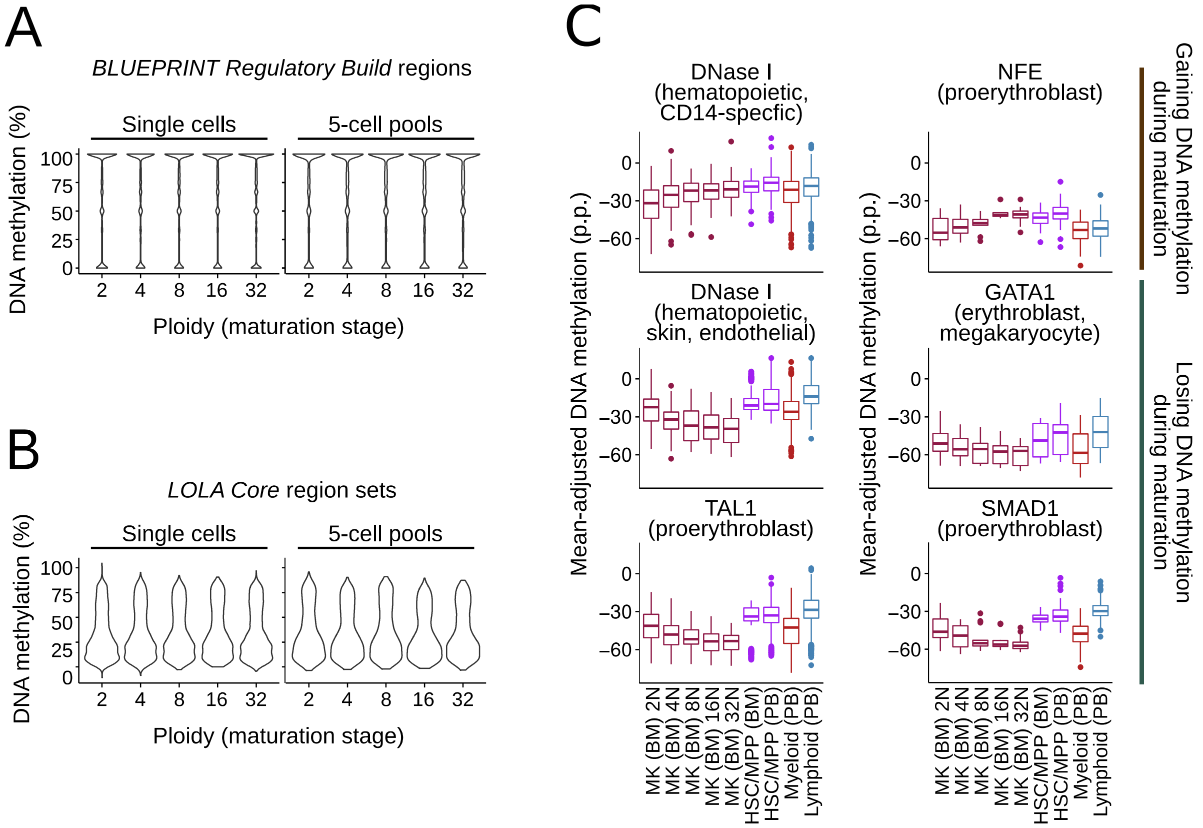

Figure S5. DNA methylation differences between megakaryocytes at different stages of ploidy

A) Violin plots showing the distribution of DNA methylation levels at BLUEPRINT Regulatory Build regions in megakaryocytes sorted according to their ploidy level (x-axis).

B) Violin plots showing the distribution of DNA methylation levels averaged across region sets in the LOLA Core database in megakaryocytes sorted according to their ploidy level (x-axis).

C) Distribution of mean-adjusted DNA methylation (relative to the average CpG methylation in each sample) across the region sets shown in **Figure 5C**. Megakaryocytes (MK) at different ploidy stages are compared to HSCs and MPPs sorted from bone marrow (BM) and peripheral blood (PB), and to myeloid progenitors (CMP, MEP, GMP) as well as lymphoid progenitors (CLP, MLP0, MLP1, MLP2, MLP3) sorted from peripheral blood.

Related to Figure 5.

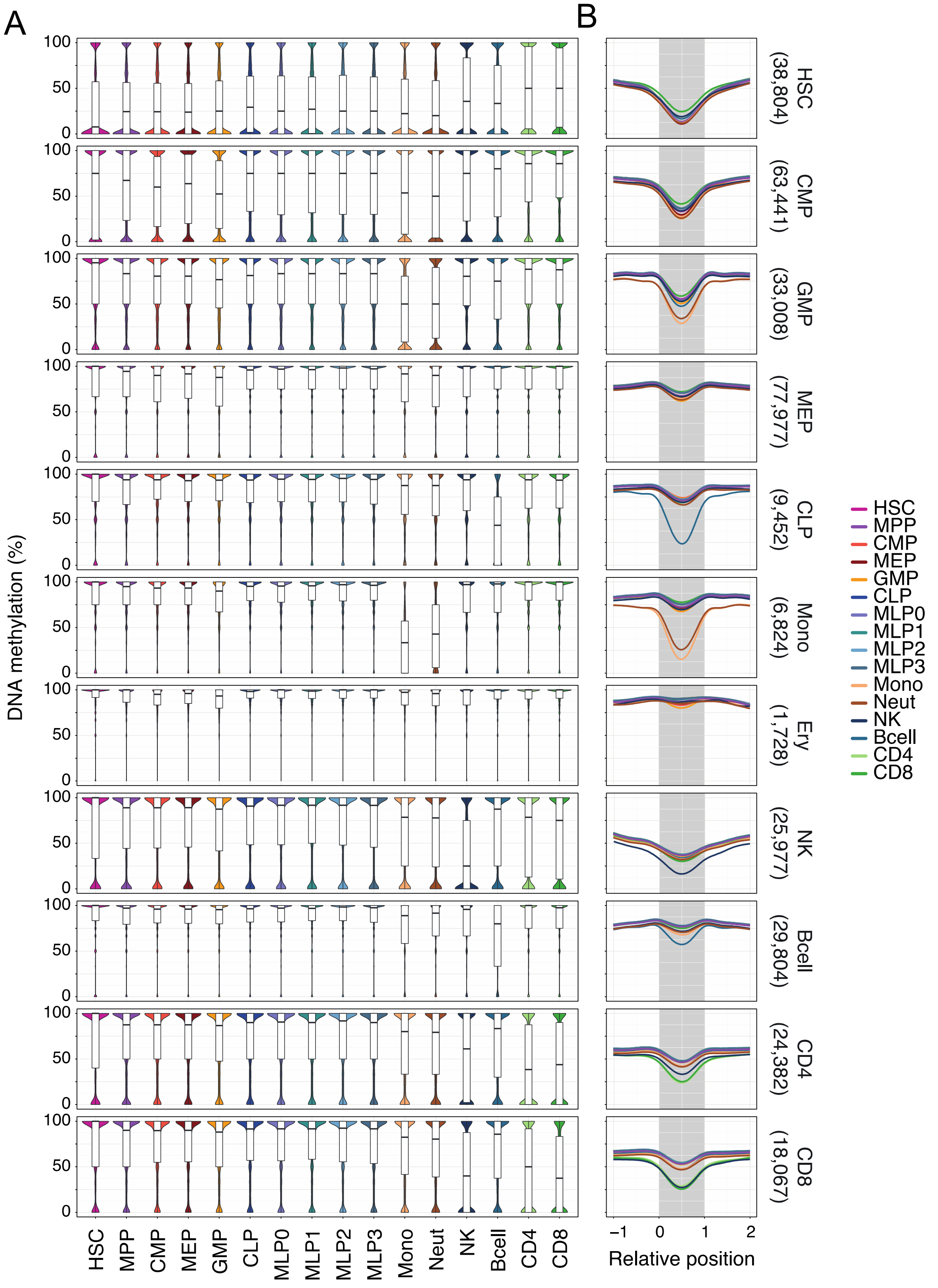

Figure S6. DNA methylation in regions with cell-type-specific chromatin accessibility

A) Boxplots showing the distribution of DNA methylation levels in regions with cell-type-specific chromatin accessibility based on published ATAC-seq data for hematopoietic cell types (GEO accession: GSE74912). The panel is an extended version of **Figure 6F**.

B) Composite plots showing DNA methylation averages across regions with cell-type-specific chromatin accessibility (numbers in parentheses). The panel is an extended version of **Figure 6G**.

Related to Figure 6.

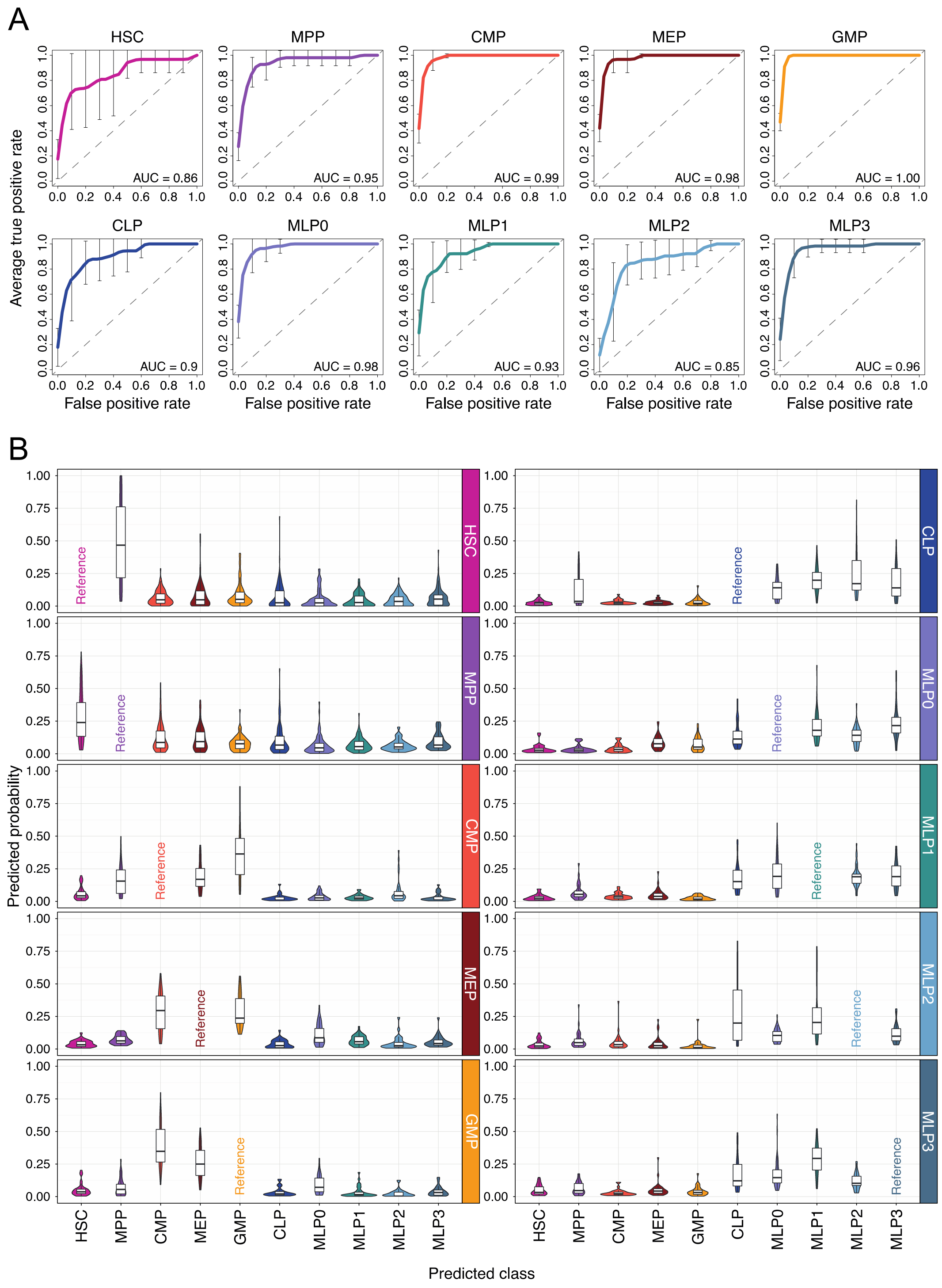

Figure S7. Prediction performance and class probability distributions for cell type classifiers

A) Receiver operating characteristic (ROC) curves and area under curve (AUC) values summarizing the prediction performance of elastic net-regularized general linear models that predict cell type from DNA methylation levels at BLUEPRINT Regulatory Build regions. The ROC curves plot the average true positive rate across 10-fold cross-validation against the false positive rate. They are based on one-versus-all prediction for each class, sliding a threshold along a value calculated as the difference of the class probability and the largest class probability excluding that class. Error bars correspond to standard deviations across the 10-fold cross-validation. Diagonal dashed lines indicate the expected performance of random guessing (AUC = 0.5).

B) Distribution of class probabilities by ten classifiers (shown in separate plots) trained on datasets that excluded all samples of one specific cell type ("leave-one-class-out classifiers").

Related to Figure 7.

Supplemental Tables

Table S1. Sample annotations and sequencing statistics

Table listing the annotation data and sequencing details for 639 DNA methylation profiles based on the µWGBS / scWGBS protocol and for 13 gene expression profiles based on the Smart-seq2 protocol.

Related to Figure 1.

Table S2. Differentially methylated regions between hematopoietic cell types and lineages

Table listing all regulatory regions from the BLUEPRINT Regulatory Build that were differentially methylated in at least one pairwise comparison between HSCs and MPPs derived from four different sources or between the myeloid and lymphoid lineages in peripheral blood. An extended version of this table with additional comparisons is available from http://blueprint-methylomes.computational-epigenetics.org.

Related to Figures 2 and 3.

Table S3. Enrichment analysis for differentially methylated regions and cell type signature regions

Region set enrichment analysis for differentially methylated regions (**Table S2**) and cell type signature regions (**Table S4**) calculated using the LOLA software tool and the LOLA Core database.

Related to Figures 2, 3, and 7.

Table S4. Signature regions identified by the cell type classifier

Table listing all regulatory regions from the BLUEPRINT Regulatory Build that contributed to the cell type classifier trained on 319 stem and progenitor samples (all 10-cell, 50-cell, and 1,000-cell pools) from peripheral blood, together with the average DNA methylation level of each region in each sample.

Related to Figure 7.

Table S5. Classifier-based similarity among the stem and progenitor cell types

Class probabilities for each stem and progenitor sample based on ten classifiers trained on datasets that excluded all samples of one specific cell type ("leave-one-class-out classifiers").

Related to Figure 7.

Supplemental Experimental Procedures

Sample collection

Peripheral blood cells were isolated from apheresis filters of healthy platelet donors belonging to the NIHR Cambridge BioResource at the NHS Blood and Transplant, Cambridge, after informed consent and with ethical approval (REC 12/EE/0040). Bone marrow for megakaryocyte sorting was obtained from otherwise healthy patients undergoing elective heart valve replacement at Barts Health NHS Trust, London, after informed consent and with ethical approval (REC 13/LO/1760). Bone-marrow-derived CD34+ cells for HSC/MPP sorting were purchased from Lonza, cat. 2M-101D (lots 0000536591, 0000476376 and 0000536050). Cord blood was collected at the Rosie Maternity Hospital, Cambridge University Hospitals, after informed consent and with ethical approval (REC 12/EE/0040). Fetal liver-derived CD34+ cells were purchased from StemExpress, cat. FL0001C (lots 1508211059, 405585112, and 1602050036).

Cell purification overview

Peripheral blood cells were extracted from apheresis filters and layered on a Ficoll-Paque gradient to isolate the fraction of mononuclear cells. After washing, the cells were processed by autoMACS (Miltenyi) to enrich for the CD34+ fraction using the posseld2 program. Cells were then stained with antibodies described below for 45 minutes at 4°C and subsequently sorted on either BD Influx or BD FACS Aria III fluorescence-activated cell sorting instruments. Bone-marrow-derived and fetal liver-derived CD34+ cells were thawed in a water bath at 37°C and resuspended in PBS1x plus DNase (10 mg/ml). After washing, the cells were stained as described above. Megakaryocytes were isolated from bone marrow as follows: A bone marrow scraping was taken after median sternotomy using a Volkmann's spoon. The sample was transported to the University of Cambridge for processing as whole bone marrow in phosphate buffered saline (PBS) containing 10% human serum albumin (HSA) and 1.8 mg/ml EDTA on ice. The cellular content was flushed out of the bone marrow using megakaryocyte buffer (PBS containing 1.2% HSA, 1.8 mg/ml EDTA), and red cells were lysed using ammonium chloride lysis. The cells were stained for megakaryocyte-specific cell surface markers with mouse APC conjugated antibody against CD41a (BD), mouse PE conjugated antibody against CD42b (BD), and for ploidy analysis with 1ug/ml Hoechst 33342 (Invitrogen). After incubation at 37°C for 30 minutes the cells were kept at 4°C before sorting using a BD FACS Aria instrument.

The cell populations were sorted using the following surface markers: HSC: Lin- CD34+ CD38- CD90+ CD45RA- CD49f+; MPP: Lin- CD34+ CD38- CD90- CD45RA- CD49f-; CMP: Lin- CD34+ CD38+ CD45RA- CD123 low; MEP: Lin- CD34+ CD38+ CD45RA- CD123- FLT3- CD36- CD110+ CD41-; GMP: Lin- CD34+ CD38+ CD45RA+ CD123+ CD10-; CLP: Lin- CD34+ CD38+ CD45RA+ CD7- CD10+; MLP0: Lin- CD34+ CD38- CD90- CD45RA+ CD7- CD10-; MLP1: Lin- CD34+ CD38- CD90- CD45RA+ CD7- CD10+; MLP2: Lin- CD34+ CD38- CD90- CD45RA+ CD7+ CD10-; MLP3: Lin- CD34+ CD38- CD90- CD45RA+ CD7+ CD10+; Megakaryocyte: CD41a+ CD42b+ Hoechst; Monocyte: CD14+ CD16- CD45+ CD64+; Neutrophil: CD16+ CD45+ CD66b+; NK cell: CD3- CD16+ CD56 low; B cell (naïve): Cd19+ CD27- IgD+; CD4 T cell (naïve): CD3+ CD4+ CD25- CD45RA+ CD62l+; CD8 T cell (naïve): CD3+ CD8+ CD25- CD45RA+ CD62L+.

Cell purification details

Isolation of CD34+ cells from apheresis filters

- Remove blood from filter into 50 ml falcon tube
- Dilute the blood up to 50 ml with room temperature Buffer 1
- Add 12.5 ml of Ficoll-Paque to two 50 ml falcon tubes
- Carefully pipette 25 ml of cell suspension on the Ficoll
- Spin 15 minutes, 800 g ↑3 ↓0
- Carefully remove the mononuclear cell layer using a 5 ml pastette
- Transfer the mononuclear cells into a fresh 50 ml tube
- Fill the tubes to 50 ml with Buffer 1 (to remove more platelets)
- Spin 6 minutes, 600 g ↑5 ↓3 (cold)
- Remove the supernatant
- Pool tubes into one 50 ml falcon tube and re-suspend in a total of 50 ml of cold Buffer 4

- Count the cells
- Spin for 6 minutes at 600 g ↑5 ↓3 (4°C)
- Remove the supernatant

Magnetic labelling and CD34+ enrichment

- Re-suspend pellet in 150µl of Buffer 4 per 10^8 cells (e.g., $9.6x10^8$ 1440 µl)
- Add 50 µl of FcR blocking reagent per 10^8 cells (e.g., $9.6x10^8$ 480 µl)
- Add 50 µl of CD34 microbeads per 10^8 cells
- Put the cells in 4°C for 30 minutes
- Add 20 ml of Buffer 4
- Spin for 6 minutes at 300 g ↑5 ↓3
- Remove supernatant
- Re-suspend pellet in 500 µl of Buffer 4 per 10^8 cells (e.g., $9.6x10^8$ 4.8 ml)
- Run sample on autoMACS using program posseld2
- Count the cells
- Stain with 1 test per 10^6 cells

Materials

- Ficoll-Paque density 1.077 (GE Healthcare, cat. 17-5442-03)
- CD34 MicroBead Kit human (Miltenyi Biotec, cat. 130-046-703) 10 ml
- PBS (Sigma, cat. D8537) 500 ml
- HAS (Gemini Bio Products, cat. 800-121)
- EDTA (Sigma, cat. E7889) 50 ml
- BSA (Sigma, cat. A9576)

Buffer 1

- PBS (Sigma, cat. D8537) 500 ml
- 1 M TriSodium Citrate 6.6 ml
- HSA 20% (0.2% final) 5 ml

Buffer 4

- PBS (Sigma, cat. D8537) 500 ml
- 0.5 M EDTA (Sigma, cat. E7889 50 ml) 2 ml (2 mM final)
- HSA 20% (0.2% final) 5 ml

Cell purification antibodies

Conjugate	Antigen	Name	Manufacturer	Product number	Concentration
**AF700**	CD3	OKT3	BioLegend	317339	5 µl/test
**AF700**	CD56	B159	BD Biosciences	557919	5 µl/test
**AF700**	CD8	SK1	BioLegend	344723	5 µl/test
**AF700**	CD14	61D3	BD Biosciences	56-0149-42	5 µl/test
**AF700**	CD11B	CBRM1/5	BD Biosciences	56-0113-42	5 µl/test
**AF700**	CD19	H1B19	BioLegend	302225	5 µl/test
**PE**	CD90	5E10	BD Biosciences	561970	5 µl/test
**PE CY 5**	CD49F	G0H3	BD Biosciences	551129	20 µl/test
**APC CY 7**	CD34	581	Molecular Probes	A14948	5 µl/test
**APC**	CD10	HI10A	BD Biosciences	332777	5 µl/test
**FITC**	CD45RA	L48	BD Biosciences	335039	20 µl/test
**PE CY 7**	CD38	HB7	BD Biosciences	335825	5 µl/test
**PB**	CD7	MT701	BD Biosciences	642916	20 µl/test
**PerCP-Cy 5.5**	CD123	7G3	BD Biosciences	560904	20 µl/test

Clonal expansion assays

Sorted single cells of the CLP, MLP0, MLP1, MLP2, and MLP3 cell populations were cultured on MS5 stroma (Itoh et al., 1989) for three weeks in conditions that support myeloid, B cell, and NK cell differentiation (Laurenti et al., 2013). Colonies were harvested, and differentiated cell types were scored by high-throughput flow cy-

tometry using the LSR II High Throughput Sampler (Becton Dickinson) with the following antibodies (Biolegend): CD45 PECy5 (1:300), CD41 FITC (1:1000), GlyA PE (BD, 1:1000), CD11b APCCy7 (1:300), CD56 APC (1:200), CD19 FITC (1:200), CD19 Alexa700 (1:300).

Whole genome bisulfite sequencing

Sequencing libraries for DNA methylation mapping were prepared using the µWGBS protocol (Farlik et al., 2015). Starting directly from lysed cells in digestion buffer, proteinase K digestion was performed at 50°C for 20 minutes. Custom-designed methylated and unmethylated oligonucleotides were added at a concentration of 0.1% to serve as spike-in controls for monitoring bisulfite conversion efficiency. Bisulfite conversion was performed using the EZ DNA Methylation-Direct Kit (Zymo Research, D5020) according to the manufacturer's protocol, with the modification of eluting the DNA in only 9 µl of elution buffer. Bisulfite-converted DNA was used for single-stranded library preparation using the EpiGnome Methyl-Seq kit (Epicentre, EGMK81312) with the described modifications (Farlik et al., 2015). Quality control of the final library was performed by measuring DNA concentrations using the Qubit dsDNA HS assay (Life Technologies, Q32851) on Qubit 2.0 Fluorometer (Life Technologies, Q32866) and by determining library fragment sizes with the Agilent High Sensitivity DNA Analysis kit (Agilent, 5067-4626) on Agilent 2100 Bioanalyzer Station (Agilent, G2939AA). All libraries were sequenced by the Biomedical Sequencing Facility at CeMM using the 2x75bp paired-end setup on the Illumina HiSeq 3000/4000 platform (see **Table S1** for sequencing statistics).

DNA methylation data processing

Sequencing adapter fragments were trimmed using Trimmomatic v0.32 (Bolger et al., 2014). The trimmed reads were aligned with Bismark v0.12.2 (Krueger and Andrews, 2011) with the following parameters: *--minins 0 --maxins 6000 --bowtie2*, which uses Bowtie2 v2.2.4 (Langmead and Salzberg, 2012) for read alignment. The GRCh38 assembly of the human reference genome was used throughout the study, in a version for sequence alignment obtained from NCBI. Duplicate reads were removed as potential PCR artefacts, and reads with a bisulfite conversion rate below 90% or with fewer than three cytosines outside a CpG context (required to confidently assess bisulfite conversion rate) were removed as potential post-bisulfite contamination. The Bismark extractor was used to estimate DNA methylation levels for each CpG. Replicates belonging to the same individual and cell type were merged by summing up the total number of methylated and unmethylated reads per CpG across all replicates. Merged and unmerged datasets were processed further using RnBeads v1.5 (Assenov et al., 2014) to generate standard analysis reports for data exploration and quality control (http://blueprint-methylomes.computational-epigenetics.org), and to aggregate DNA methylation values of individual CpGs based on genomic tiling regions (width: 5 kilobases) and based on regulatory regions annotated by the August 2015 release of the BLUEPRINT Ensembl Regulatory Build (Zerbino et al., 2015). The DNA methylation tables produced by RnBeads were the basis for further data analysis with custom R scripts.

Differential DNA methylation analysis

We analyzed differential DNA methylation for regulatory regions defined by the August 2015 release of the BLUEPRINT Ensembl Regulatory Build (**Figure 2B, 3A, Table S2**). All pairwise comparisons were performed with the differential methylation module in RnBeads (Assenov et al., 2014), which uses the limma method for statistical analysis (Ritchie et al., 2015). Potential confounding factors such as flowcell, gender, and number of cells sequenced were statistically accounted for in the RnBeads analysis. Regions were considered differentially methylated if they had an FDR-adjusted p-value below 0.05 and an absolute change in DNA methylation that was among the top 5% strongest absolute differences observed across all pairwise comparisons (which corresponds to a difference in absolute DNA methylation levels of at least 16.7 percentage points). We further removed regions that had not been covered in at least three samples and regions that had not been covered with at least three reads in at least one sample.

Region set enrichment analysis

We used LOLA (Sheffield and Bock, 2015) to identify significant overlaps of differentially methylated regions and cell type signature regions with empirically defined transcription factor binding sites based on ChIP-seq datasets obtained from ENCODE (Harrow et al., 2012) and from the CODEX database (Sánchez-Castillo et al., 2015). Fisher's exact test was used with a significance threshold of 0.05 on Benjamini-Yekutieli adjusted p-values. Figure panels include all transcription factors that were enriched in at least one of the relevant comparisons, while also showing enrichment data for these transcription factors in cell types where they were not enriched. To facilitate visualization and interpretation, we manually grouped the annotations into broader categories. All enriched results together with their original and curated annotations are available in **Table S3**. ChIP-seq datasets for malignant cell populations were excluded from the figures given the study's focus on normal hematopoietic differentiation (but they are included in **Table S3**).

Single-cell DNA methylation analysis

To compensate for the sparseness of low-input and single-cell DNA methylation data, several analyses (**Figure 3D-G**, **S3B-D**, **4B**, **4C**, **5B**, **5C**, **S5B**, **S5C**) employed a region set analysis strategy described previously (Farlik et al, 2015). This bioinformatic method is based on the observation that characteristic cell-type-specific DNA methylation differences can be identified by calculating average DNA methylation levels across sets of functionally related regions (e.g., across binding sites of a transcription factor or enhancer elements active in a certain cell type). We used the LOLA Core database (Sheffield & Bock, 2015), a large catalog of experimentally identified regulatory region sets, as our reference. For each stem and progenitor dataset we calculated average DNA methylation levels across each region set. We adjusted these values for differences in global DNA methylation levels between cell types by subtracting, in each sample, the global DNA methylation average across all CpGs from the region set values. Analyses based on individual low-input and single-cell samples used these region set estimates of mean-adjusted DNA methylation (relative to the average CpG methylation level in each sample) in the same way as analyses based on pooled replicates used region-level DNA methylation data for the BLUEPRINT Regulatory Build. We used Wilcoxon rank sum tests and considered region sets with a p-value ≤ 0.05 and an absolute change in mean-adjusted DNA methylation of at least 10 percentage points as differentially methylated.

RNA sequencing

Cells were sorted directly into lysis buffer containing 0.2% Triton X-100 and RNase inhibitor. The cDNA synthesis and poly(A) enrichment were performed following the Smart-seq2 protocol (Picelli et al., 2014). ERCC spike-in RNA (Ambion) was added to the lysis buffer in a dilution of 1:1,000,000. Library preparation was performed on 0.5 ng cDNA using the Nextera XT library preparation kit (Illumina) following the manufacturer instructions. All libraries were sequenced by the Biomedical Sequencing Facility at CeMM using the 1x50 bp single-read setup on the Illumina HiSeq 3000/4000 platform (see **Table S1** for sequencing statistics).

Gene expression analysis

Sequencing adapter fragments were trimmed using Trimmomatic v0.32 (Bolger et al., 2014). The trimmed reads were aligned to the cDNA reference transcriptome (GRCh38 cDNA sequences from Ensembl) using Bowtie v1.1.1 (Langmead et al., 2009) and the following parameters: *-q -p 6 -a -m 100 --minins 0 --maxins 5000 --fr --sam --chunkmbs 200*. Duplicate reads were removed, and transcript levels were quantified with BitSeq v1.12.0 (Glaus et al., 2012). Transcript-level expression estimates were loaded into R and collapsed into gene-level estimates by using the most highly expressed transcript variant. DESeq2 (Love et al., 2014) was used for statistical analysis of the read counts. Genes with an FDR-corrected p-value ≤ 0.05 and at least a two-fold change in expression ($|\log_2 FC| \geq 1$) were considered as differentially expressed. Gene expression estimates for visualization and reporting were adjusted by variance stabilization.

Integration of histone modification data

We processed all histone data of the September 2015 BLUEPRINT release (seventh data release) using a similar approach as in the Roadmap Epigenomics analysis (Ernst and Kellis, 2015; Kundaje et al., 2015). Briefly, we selected all samples for which the input control and at least three of the six histone modifications (H3K27ac, H3K27me3, H3K36me3, H3K4me1, H3K4me3, H3K9me3) were available and generated genome-wide tracks for the ChIP-seq signal enrichment over input using MACS2 v2.1.0 (Zhang et al., 2008). These tracks were used as input to ChromImpute v1.0.0 (Ernst and Kellis, 2015), imputing all missing data and merging replicates. The p-values calculated by MACS2 were used as intensity estimates for the boxplots.

Integration of open chromatin data

We downloaded peak regions and fragment counts from ATAC-seq experiments (GEO accession GSE74912) for hematopoietic cell types (Corces et al., 2016) and transformed the peak coordinates to genome assembly GRCh38 using the UCSC liftOver tool (https://genome.ucsc.edu/cgi-bin/hgLiftOver). Average DNA methylation levels across samples were computed for all ATAC-seq peaks. We used the one-sided Wilcoxon rank sum test to identify cell-type-specific regions of open chromatin. Specifically, for each cell type in the ATAC-seq data set we selected those peak regions in which samples of that cell type exhibited a significantly higher ATAC-seq fragment count than samples not belonging to that cell types (FDR adjusted p-value ≤ 0.05).

Cell type prediction

Samples were classified using elastic net-regularized general linear models as implemented in the R package *glmnet* (Friedman et al., 2010; Krishnapuram et al., 2005). Classifiers were trained on 319 stem and progenitor samples (all 10-cell, 50-cell, and 1,000-cell pools) from peripheral blood, using their DNA methylation profiles across regulatory regions from the BLUEPRINT Ensembl Regulatory Build as prediction variables. Missing values were imputed with the *impute* R package (https://bioconductor.org/packages/release/bioc/html/impute.html) using 5-nearest neighbor averaging. Elastic net regularization was applied to a multinomial logistic regression classifier. The regularization parameter λ was obtained by nested 10-fold cross-validation, and α was set to 0.5 to stipulate equal mixing of the lasso and ridge penalty terms. Class importance was defined as the L2 norm aggregate of per-class coefficients in the model. Class probabilities were defined as fitted probabilities from the logistic regression model. For assessing model quality, 10-fold cross validation was performed and misclassification rates were averaged over the cross-validation test sets. Per-class ROC curves and area under curve (AUC) values were obtained by evaluating the class probabilities in the one-versus-all setting for each class, i.e., by sliding a threshold along the score resulting from the difference of the class probability and the largest class probability excluding that class. Signature regions were defined as those regulatory regions that were assigned a non-zero class importance value in the model trained on the entire dataset (**Table S4**). For quantifying class probabilities of individual cell types (**Figures S7B, Table S5**), the samples of one class were excluded from the training, and the resulting model applied to the samples excluded from training ("leave-one-class-out classifiers").

Inference of cell type similarity graph

In the cell type similarity graph (**Figure 7F**), nodes represent cell types and edges represent probabilities of predicting one cell type as another using the corresponding "leave-one-class-out" classifier. Specifically, for each pair of source and target cell type, the edge weight is the average class probability assigned by the leave-one-class-out classifier to all peripheral blood samples of the source cell type to the target cell type. The graph shows the directed edge pairs for each pair of nodes as trapezoids in which the widths at the target and source node correspond to weights of the directed edges. For example, the predictor that did not include HSCs assigned a higher probability to classify HSCs as MPPs than the probabilities the predictor which did not include MPPs assigned to predicting MPPs as HSCs. Differentiated cell types (circles) were predicted based on the classifier trained on all 319 stem and progenitor samples from peripheral blood and are connected by grey edges in the graph. Edges with a prediction probability below 0.1 were pruned. The graph layout was automatically generated using the Fruchterman-Reingold algorithm as implemented in the R package *igraph*.

Supplemental References

Assenov, Y., Müller, F., Lutsik, P., Walter, J., Lengauer, T., and Bock, C. (2014). Comprehensive analysis of DNA methylation data with RnBeads. Nat Methods *11*, 1138-1140.

Bolger, A.M., Lohse, M., and Usadel, B. (2014). Trimmomatic: a flexible trimmer for Illumina sequence data. Bioinformatics *30*, 2114–2120.

Corces, M.R., Buenrostro, J.D., Wu, B., Greenside, P.G., Chan, S.M., Koenig, J.L., Snyder, M.P., Pritchard, J.K., Kundaje, A., Greenleaf, W.J., *et al.* (2016). Lineage-specific and single-cell chromatin accessibility charts human hematopoiesis and leukemia evolution. Nat Genet 48, 1193–1203.

Ernst, J., and Kellis, M. (2015). Large-scale imputation of epigenomic datasets for systematic annotation of diverse human tissues. Nat Biotechnol *33*, 364–376.

Farlik, M., Sheffield, N.C., Nuzzo, A., Datlinger, P., Schönegger, A., Klughammer, J., and Bock, C. (2015). Single-cell DNA methylome sequencing and bioinformatic inference of epigenomic cell-state dynamics. Cell Rep *10*, 1386-1397.

Friedman, J., Hastie, T., and Tibshirani, R. (2010). Regularization Paths for Generalized Linear Models via Coordinate Descent. J Stat Softw *33*, 1–22.

Glaus, P., Honkela, A., and Rattray, M. (2012). Identifying differentially expressed transcripts from RNA-seq data with biological variation. Bioinformatics *28*, 1721-1728.

Harrow, J., Frankish, A., Gonzalez, J.M., Tapanari, E., Diekhans, M., Kokocinski, F., Aken, B.L., Barrell, D., Zadissa, A., Searle, S., et al. (2012). GENCODE: The reference human genome annotation for The ENCODE Project. Genome Res *22*, 1760–1774.

Itoh, K., Tezuka, H., Sakoda, H., Konno, M., Nagata, K., Uchiyama, T., Uchino, H., and Mori, K.J. (1989). Reproducible establishment of hemopoietic supportive stromal cell lines from murine bone marrow. Exp Hematol *17*, 145-153.

Krishnapuram, B., Carin, L., Figueiredo, M.A.T., and Hartemink, A.J. (2005). Sparse multinomial logistic regression: fast algorithms and generalization bounds. IEEE Trans Pattern Anal Mach Intell *27*, 957–968.

Krueger, F., and Andrews, S.R. (2011). Bismark: a flexible aligner and methylation caller for bisulfite-seq applications. Bioinformatics *27*, 1571-1572.

Kundaje, A., Meuleman, W., Ernst, J., Bilenky, M., Yen, A., Heravi-Moussavi, A., Kheradpour, P., Zhang, Z., Wang, J., et al. (2015). Integrative analysis of 111 reference human epigenomes. Nature *518*, 317–330.

Langmead, B. and Salzberg, S.L. (2012). Fast gapped-read alignment with Bowtie 2. Nat Methods *9*, 357-359.

Langmead, B., Trapnell, C., Pop, M., and Salzberg, S.L. (2009). Ultrafast and memory-efficient alignment of short DNA sequences to the human genome. Genome Biol *10*, R25.

Laurenti, E., Doulatov, S., Zandi, S., Plumb, I., Chen, J., April, C., Fan, J.B., and Dick, J.E. (2013). The transcriptional architecture of early human hematopoiesis identifies multilevel control of lymphoid commitment. Nat Immunol *14*, 756-763.

Love, M.I., Huber, W., and Anders, S. (2014). Moderated estimation of fold change and dispersion for RNA-seq data with DESeq2. Genome Biol *15*, 550.

Picelli, S., Faridani, O.R., Bjorklund, A.K., Winberg, G., Sagasser, S., and Sandberg, R. (2014). Full-length RNA-seq from single cells using Smart-seq2. Nat Protoc *9*, 171-181.

Ritchie, M.E., Phipson, B., Wu, D., Hu, Y., Law, C.W., Shi, W., and Smyth, G.K. (2015). limma powers differential expression analyses for RNA-sequencing and microarray studies. Nucleic Acids Res *43*, e47.

Sánchez-Castillo, M., Ruau, D., Wilkinson, A.C., Ng, F.S.L., Hannah, R., Diamanti, E., Lombard, P., Wilson, N.K., and Göttgens, B. (2015). CODEX: a next-generation sequencing experiment database for the haematopoietic and embryonic stem cell communities. Nucleic Acids Res. *43*, D1117–D1123.

Sheffield, N.C., and Bock, C. (2016). LOLA: enrichment analysis for genomic region sets and regulatory elements in R and Bioconductor. Bioinformatics *32*, 587-589.

Zerbino, D.R., Wilder, S.P., Johnson, N., Juettemann, T., and Flicek, P.R. (2015). The Ensembl Regulatory Build. Genome Biol. *16*, 56.

Zhang, Y., Liu, T., Meyer, C.A., Eeckhoute, J., Johnson, D.S., Bernstein, B.E., Nusbaum, C., Myers, R.M., Brown, M., Li, W., et al. (2008). Model-based analysis of ChIP-Seq (MACS). Genome Biol. *9*, R137.
